# Detection and identification of transgenic events by next generation sequencing combined with enrichment technologies

**DOI:** 10.1038/s41598-019-51668-x

**Published:** 2019-10-30

**Authors:** Frédéric Debode, Julie Hulin, Benoît Charloteaux, Wouter Coppieters, Marc Hanikenne, Latifa Karim, Gilbert Berben

**Affiliations:** 1 Walloon Agricultural Research Center (CRA-W), Unit Traceability and Authentication, chaussée de Namur 24, 5030 Gembloux, Belgium; 2 University of Liège, GIGA - Genomics Platform, B34, 4000 Liège (Sart Tilman), Belgium; 3 University of Liège, InBioS - PhytoSystems, Functional Genomics and Plant Molecular Imaging, Chemin de la Vallée, 4, B22, 4000 Liège (Sart Tilman), Belgium

**Keywords:** Sequencing, Molecular biology

## Abstract

Next generation sequencing (NGS) is a promising tool for analysing the quality and safety of food and feed products. The detection and identification of genetically modified organisms (GMOs) is complex, as the diversity of transgenic events and types of structural elements introduced in plants continue to increase. In this paper, we show how a strategy that combines enrichment technologies with NGS can be used to detect a large panel of structural elements and partially or completely reconstruct the new sequence inserted into the plant genome in a single analysis, even at low GMO percentages. The strategy of enriching sequences of interest makes the approach applicable even to mixed products, which was not possible before due to insufficient coverage of the different genomes present. This approach is also the first step towards a more complete characterisation of agrifood products in a single analysis.

## Introduction

The number and diversity of GMOs have greatly increased in recent years. Currently, the reference method for GMO detection is real-time PCR. The main problem of real-time PCR is that it can only be used to detect targeted sequences, which means that searches are somewhat limited since they can only find what is being looked for. Moreover, new solutions need to be found for the characterisation of authorised and unauthorised GMOs. NGS approaches may address the problem of identifying all GMOs in a sample. High-throughput sequencing can sequence several million fragments in parallel and is able to provide the whole sequence of plant genomes^1^. NGS has already been used to help molecularly characterise a genetically modified (GM) soybean without the need for Southern Blot analysis^2^. Several approaches have been developed that use the potential of high-throughput sequencing for the detection of GMOs or GMO-derived products^3–6^. However, NGS is still not frequently used for GMO detection due to important challenges, such as uneven coverage of the genome^7^. This problem can be reinforced as a function of the genome size of the plant considered, e.g., the soybean genome is ~1.1 gigabases (Gb)^8^ while the wheat genome is ~17 Gb^9^, and genetic diversity is even greater in complex food products containing several plant species.

Several approaches for GMO detection have already been developed. First, pilot studies have shown that NGS using whole genome sequencing approaches is able to detect GMOs^10–12^. NGS became a method for checking for inserted sequences^2–4^. However, these methods have only been tested on pure GM material, while a large number of sequencing runs would be required to gain sufficient coverage to allow the detection of low GM contents^7^. To detect GMOs present at low levels, sequencing of a large number of targeted amplicons by NGS was proposed^13^. This method was able to detect numerous structural elements but was not suitable for reconstructing the inserted sequence. The sensitivity of this method was not evaluated in depth, but its performance was poorer than real-time PCR^13^. Only techniques combining NGS with SiteFinding PCR^5^ and DNA walking strategies^14,15^ have been able to provide information on the junction sequence between a plant and GM construct at low percentages. The method using genome walking with ALF (amplification of linearly enriched fragment) could detect a level as low as 1%^15^. This method starts with two structural elements, p35S and tNOS. DNA walking method using anchored PCR followed by two semi-nested PCRs was able to detect a level of 0.1% of Bt rice^14^. This method is now capable of starting from five structural elements (p35S, t35S pCambia, tNOS and cry)^14,16^. However, these strategies, based on the sequencing of amplicons by NGS, are time-consuming, cannot cover large fragments of GM constructs and are dependent on a starting point linked to the presence of a precise structural element.

We developed an approach combining NGS with a strategy of enriching the regions of interest that differs from the eleven enrichment strategies listed by Arulandhu *et al*.^17^. The regions of interest correspond to a series of structural elements frequently introduced into transgenic constructs. We checked the capacity of the method for GMO detection, even in flour containing low percentages of transgenic plants, and developed a bioinformatic pipeline for the detection and characterisation of GM events.

## Results and Discussion

Our work started with the development of a database of sequences that could be used for enrichment. The present version of the database gathers the sequences of 10 promoters, 6 terminators and 23 genes or miscellaneous elements that are found in transgenic constructs (Table 1). The total size of the enrichment sequences in the database is ~53 kb, but the database is still far from its limit as the methodology can be scaled up to 24 Mb. The covering a large number of GM events is possible, as the database includes the sequences of the structural elements most commonly used in genetically modified plants^18,19^. Sequences corresponding to antibiotic resistance or other selection markers were not included in the database, as they could generate unexpected signals linked to the presence of traces of DNA from the bacteria and recombinant plasmids used for the production of the enzymes employed for PCR amplification and sequencing. If we compare the potential of detection with the 328 GM events listed in the GMOseek matrix^19^ and in relation to 23 plant species, only 3 GM events (AR9 Azuki bean, LY038 maize and BPS-CV127-9 soybean) would not be detected because they do not contain any of the 40 structural elements used to design the enrichment. AR9 Azuki bean, LY038 maize and BPS-CV127-9 soybean contain structural elements that are particular to these transgenic events. The sequences of these structural elements are not currently available but could be added in the future. However, the AR9 Azuki bean also contains *npt*II, providing tolerance to antibiotics^20^. This example shows the importance of not excluding selection markers from the enrichment database in the future and is why the pros and cons of the presence of such sequences should be evaluated in the next version of the enrichment database.

**Table 1:** List of the structural elements used for the enrichment step.

Type of structural element	Name	Size (bp)	Sequence source
Promoters	p35S	867	NCBI KX880509
	pFMV	981	NCBI X06166
	pUbi	2018	NCBI S94464
	pNOS	398	Patent WO2006074956
	pmas	660	NCBI DQ225747
	Ps7s7	1046	NCBI AY181091
	pRice actin1	660	NCBI S44221
	pRice actin2	259	NCBI EU161577
	pSSuAra	1727	NCBI CP002684
	pTA29	627	NCBI X52283
	pMTL	2556	NCBI S57628
Terminators	tOCS	823	NCBI LT727071
	tE9	648	Patent W02007027777
	tNOS	300	NCBI AB809952
	tg7	203	Patent WO2006074956
	tpinII	318	NCBI KP784700
	t35S	211	NCBI GU734649
Genes	gus	576	NCBI CP029981
	gox	1296	Patent US5463175
	cryIAb 1	1854	Patent US20030226171
	cryIAb 2	3844	NCBI AY326434
	cryIAb 3	1947	Patent US5625136
	cry1B	1950	NCBI KC414884
	cry1Aa	1848	NCBI GU583855
	cry1Ab/c	1923	NCBI GU583854
	cry1Ac	1923	NCBI KF630361
	cry1A105 1	3537	Patent WO200702777
	cry1A105 2	3433	NCBI DI362404
	cry2Ab2	1900	NCBI DI362404
	cry3A055	1797	Patent EP2289311
	cry34Ab1	424	Patent W02006039376
	cry35Ab1	981	Patent W02006039376
	EPSPS 1	1415	NCBI AB209952
	EPSPS 2	1367	Patent WO2004074492
	2mEPSPS	1338	Patent WO2011063411
	bar	835	NCBI X05822
	pat	569	NCBI GQ497217
	Prsv-cp 1	1601	NCBI F5490192
	Prsv-cp 2	1070	NCBI GZ450610
Miscellaneous	hsp70	804	NCBI AY326434
Total length of the database used for enrichment		52534	

When several variants of the same gene were present, they were identified with a different numeric index (e.g., EPSPS1, EPSPS2).

The developed database was then used to create capture probes focusing on the elements listed in Table 1. Two types of methodologies were tested for sequence enrichment through capture probes. The first methodology used numerous probes of 50-80 bp that had a high level of overlap (SeqCapEZ technology, Roche Diagnostics/NimbleGen, Madison, WI), in which each base is generally covered by at least 7 probes. The second methodology used larger probes (~120 bp) with a low level of overlap (SureSelect technology, Agilent Technologies, Santa Clara, CA). No degeneracy was introduced in the probes. The probes are supposed to be able to catch fragments of up to 500 bp in size, which would allow the captured fragments to include junctions between structural elements or junctions between the plant and inserted sequence.

**Figure 1:**
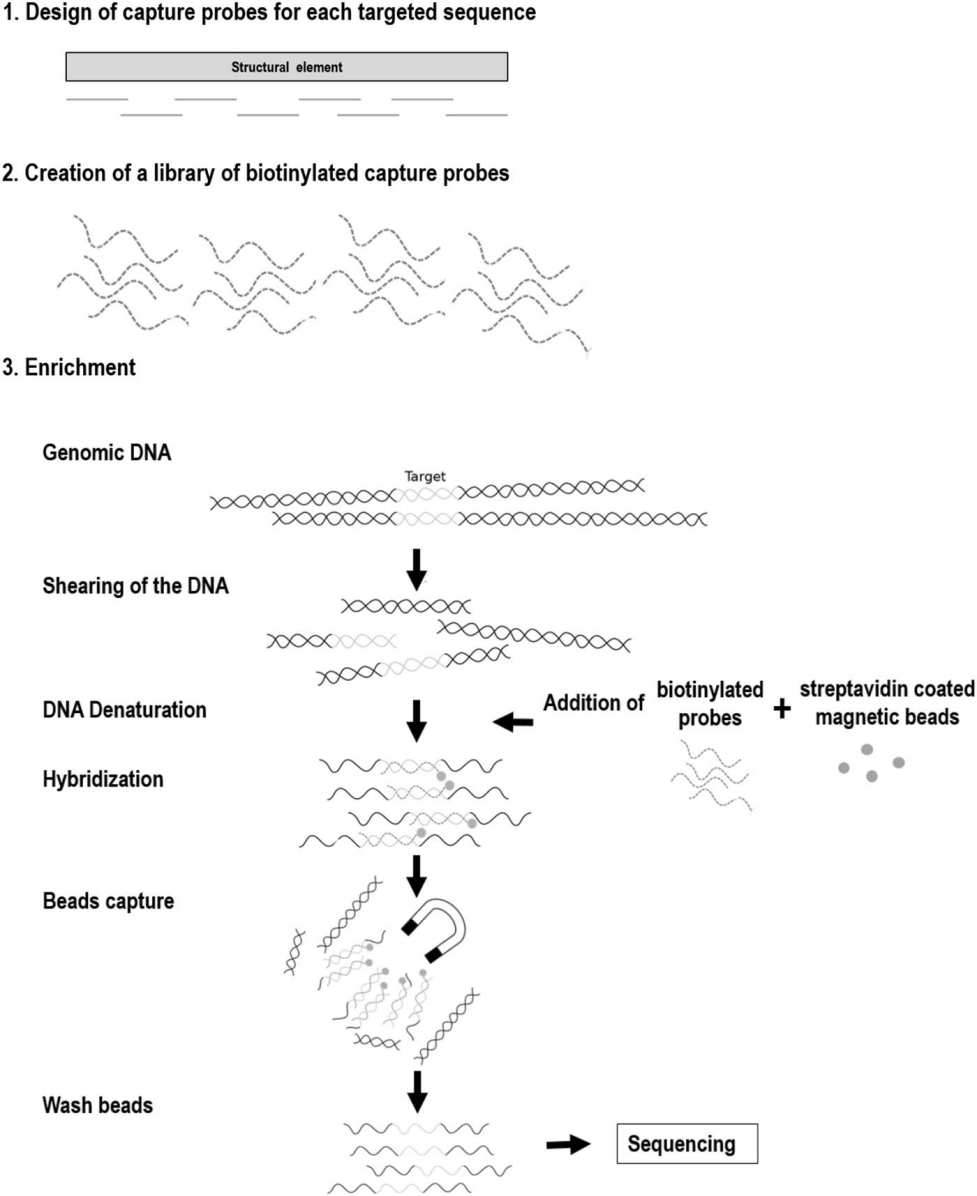
Workflow of the enrichment technology prior to sequencing.

The enrichment principle is presented in Fig. 1. From a theoretical point of view, both methodologies have advantages: shorter and more numerous probes should be better at capturing degraded DNA or sequences of structural elements that slightly vary from what is expected, while longer probes should lead to increased specificity of sequence capture. The comparison of the SureSelect and NimbleGen technologies has already been discussed for several medical applications with results favouring either the NimbleGen approach^21–23^ or SureSelect technology^24^ or indicating comparable performances^25^. The comparisons show that both methodologies have pros and cons depending on the objectives of the project^26^ and indicate that the balance in favour of one method can change as a function of the evolution of kits and protocols^26^.

In this study, after analysing sequencing runs on Illumina devices, better enrichments with fewer unexpected assignations were observed when using SureSelect technology. This paper focuses on the best results obtained with this technology. After enrichment, the DNA libraries were sequenced on an Illumina MiSeq system (Illumina, San Diego, CA).

**Figure 2:**
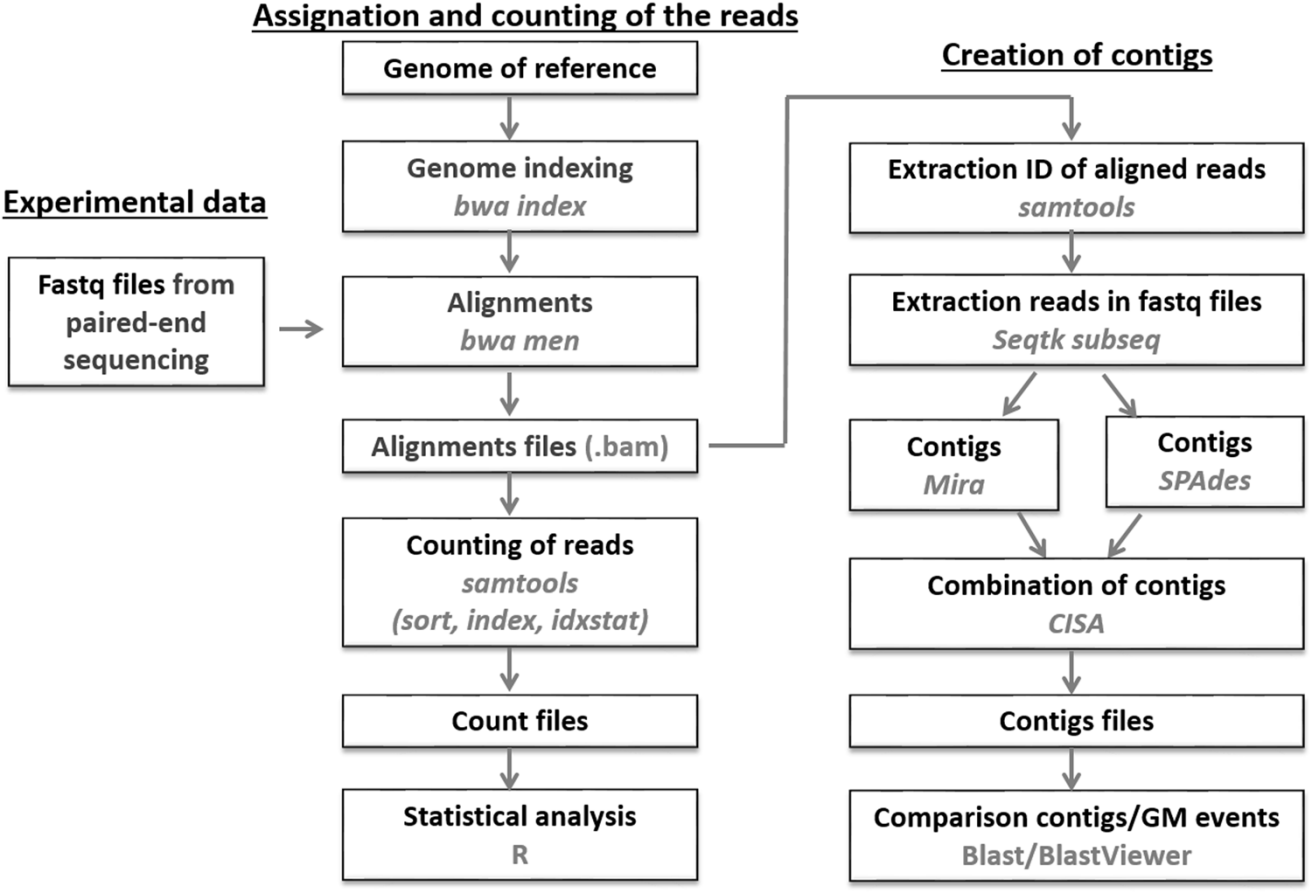
Bioinformatic workflow developed for detecting and identifying GMOs. The bioinformatic packages used are indicated in grey.

To analyse the large amount of read data, a bioinformatic workflow was created. The workflow was divided into two parts. In the first part, which was aimed at GMO detection, reads were aligned onto the sequences used for enrichment and filtered according to their alignment scores. Statistical analysis was then performed to determine whether the reads could be distinguished from noise and assimilated to positive results. The objective of the second part of the workflow was to characterise the GMO through the creation of contigs in an attempt to reconstruct the whole transgene, possibly including the plant-construct junction specific to the event. The bioinformatics workflow used different scripts and programs, as presented in Fig. 2.

**Figure 3:**
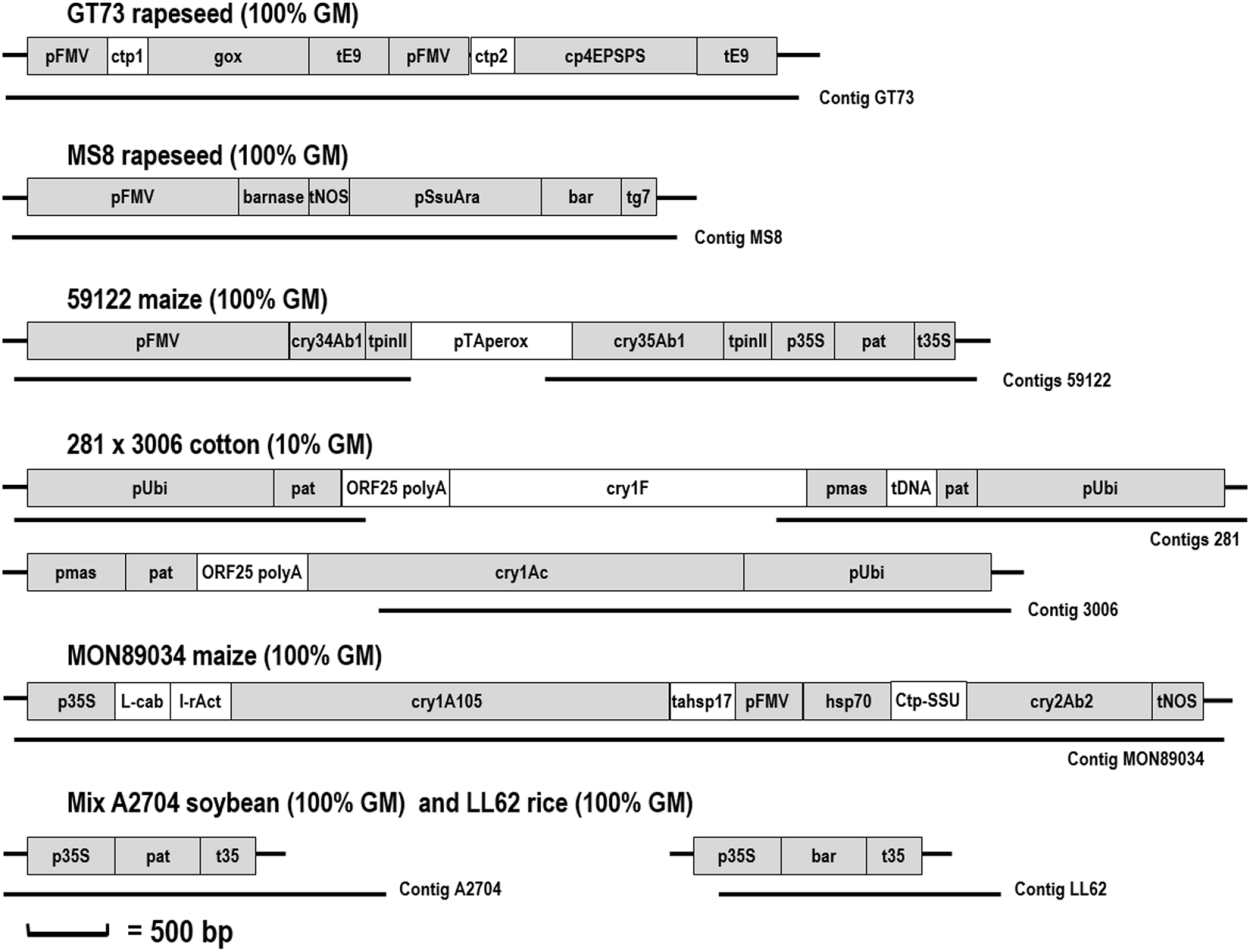
Detection and characterisation of GMOs by NGS. The structures of the inserts of seven GMOs are presented. The 281 × 3006 cotton has two GM inserts. The mixed sample contains 50% A2704 soybean and 50% LL62 rice. The structural elements in grey were present in the database used for enrichment and were detected by NGS. The reads associated with these structural elements were used to create contigs. Only larger contigs covering several structural elements are shown here. Larger structural elements not covered by the capture probes created gaps, making it impossible to reconstruct the entire sequence of the transgenic cassette. Junction regions covering the plant and transgenic insert were also obtained.

**Figure 4:**
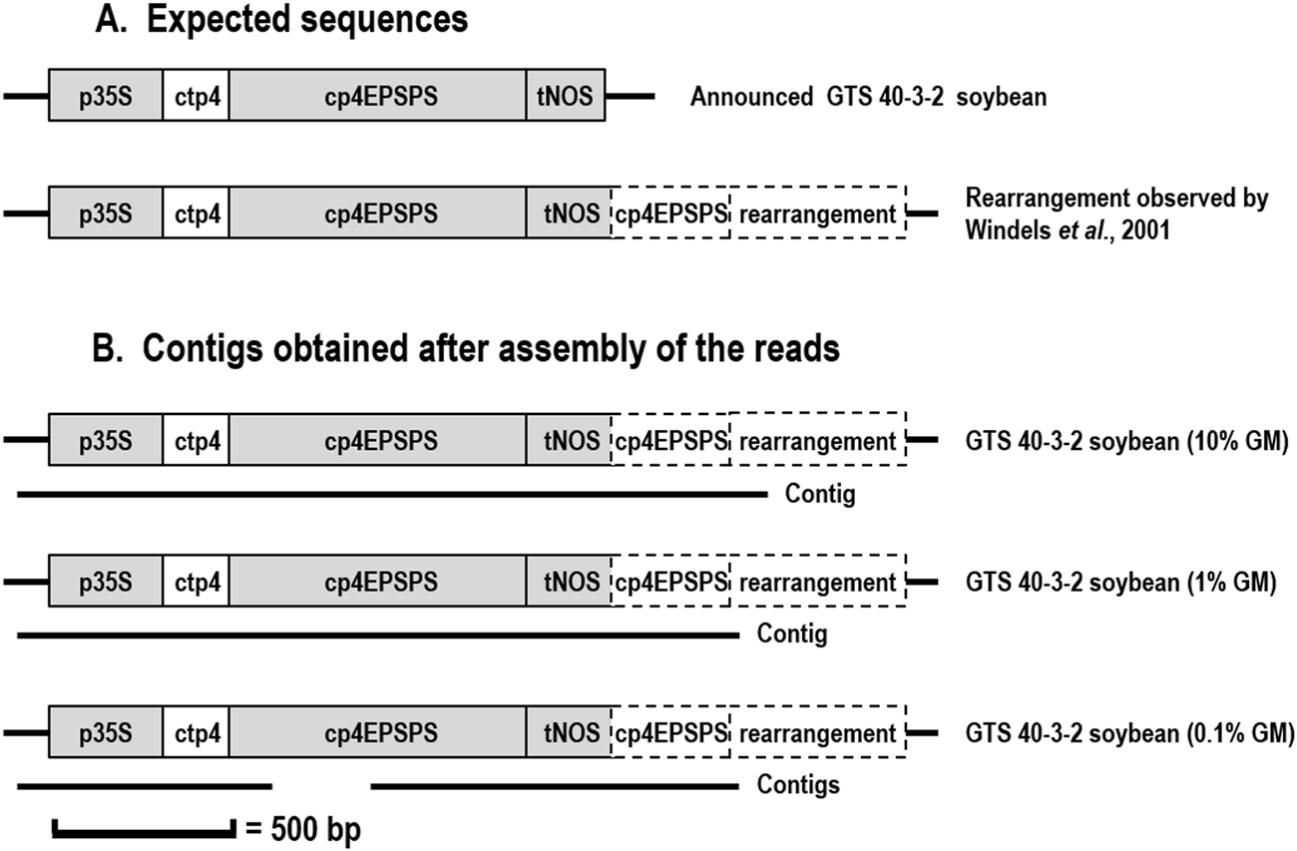
Sequence of GTS 40-3-2 soybean and alignments of the contigs obtained in this research. The structural elements in grey shown in the database were used for enrichment and were detected by NGS. (**A**) Expected sequences of the GTS-40-3-2 soybean, as announced by Monsanto and as described by Windels *et al*.^28^. Additional sequence corresponds to a duplication of part of the EPSPS gene and an unknown rearranged sequence. (**B**) Positions of the contigs created for the samples containing GTS-40-3-2 soybean at 10%, 1% and 0.1%.

The analysed samples included five species, eight transgenic events and variable fractions (0.1%, 1%, 10% and 100%) of GMOs (Table 2 in the methods section). Concerning GMO detection, the structural elements listed in the enrichment database and present in the GM events tested were all detected (Figs 3 and 4).

**Table 2:** Origin of the samples used for analysis by NGS.

Material used	Reference	Provider
Rapeseed GT73 (100% GM)	AOCS 0304-B	AOCS
Rapeseed MS8 (100% GM)	AOCS 0306-F2	AOCS
Maize 59122 (10% GM)	ERM-BF424d	IRMM
Cotton 281 × 3006 (10% GM)	ERM-BF422d	IRMM
Maize MON89034 (100% GM)	AOCS 0906-E	AOCS
Soybean A2704-12 (100% GM)	AOCS 0707-B4	AOCS
Rice LL62 (100% GM)	AOCS 0306-I4	AOCS
Soybean GTS 40-3-2 (0.1% GM)	ERM-BF410bk	IRMM
Soybean GTS 40-3-2 (1% GM)	ERM-BF410dk	IRMM
Soybean GTS 40-3-2 (10% GM)	ERM-BF410gk	IRMM
Maize (0% GM)	Commercial organic maize	(Ekibio, Peaugres, France)
Soybean (0% GM)	Commercial organic soybean	(Ekibio, Peaugres, France)

Logically, the percentage of sequenced reads assigned to the structural element present depended on the GM percentage. An example is given in Table 3 for GTS-40-3-2 soybean, in which the percentage of reads aligned with p35S, tNOS and EPSPS increased as a function of the GM percentage. The absolute number of reads is linked to the length of the structural elements (this point can, however, be normalised) and to the DNA quantities introduced in the experiments. The number of reads cannot be used for quantitative approaches, and sequencing will not replace real-time PCR or digital PCR for GMO quantification. However, once the system is updated with taxon-specific genes (preliminary experiments are underway), the system may be able to provide an indication of the GM percentage. This information would, however, remain semi-quantitative.

**Table 3:** Reads obtained in soybean flour containing 0.1%, 1% and 10% GTS 40-3-2 soybean (results obtained with reads of 75 bp; the number of reads was not normalised to the length of the structural element considered here).

Reads	Soybean 0% GM	Soybean GTS 40-3-2 0.1% GM	Soybean GTS 40-3-2 1% GM	Soybean GTS 40-3-2 10% GM
Number of reads	2103154	2098020	2226922	1961322
Number of reads aligned	211	1423	11023	74739
% of reads aligned	0.01	0.07	0.49	3.81
Reads aligned with p35S	0	128	1275	10504
Reads aligned with tNOS	4	142	1438	8743
Reads aligned with EPSPS	28	824	8107	55316

The values presented in the table are the results of a single analysis.

In the bioinformatics workflow, a threshold level was set for considering an element beyond background noise and thus as being detected. In the SureSelect experiments, this threshold was based on the mean number of reads, standardised in RPKM (reads per kilobase per million mapped reads), obtained for non-GM plants plus five times the calculated standard deviation, giving a probability of differentiation between positive and negative results greater to 99%^27^. Structural elements were clearly distinguished from each other except for *cry* gene sequences, as its variants showed similarities in their sequences. However, the highest number of assignations was attributed to the correct cry gene. Non-GM plants were also tested to check for unspecific mappings. With a threshold of 25 reads (standardised in RPKM), no problems were encountered with soybean, cotton or rapeseed. For maize, positive signals were observed with pUbi, pMTL and hsp70, as maize is the donor organism of these structural elements. Some similarities were also identified in maize for the EPSPS1 structural element (GACGAGGAAGCTCATGGCGATGCGGTGATCGAGATGGGTGGCGACG), as this element showed similarity with a 46-bp fragment of the maize genome. Information concerning the donor organism of the structural element and its potential presence in the sample must be taken into account to interpret the results, but the element could also be a target of interest for implementation of the detection system for the identification of plants.

For GMO characterisation, positive reads were assembled to create “blind” contigs to prevent influence from a previously known sequence. This process is important for detecting differences between the announced and the real sequence of a GMO and to mimic results that could be obtained in the presence of an unknown GMO. Contigs made it possible to partially (100% 59122 maize and 10% 281 × 3006 cotton) or totally (10% GTS-40-3-2 soybean, 100% GT73 rapeseed, 100% MS8 rapeseed, 10% MON89034 maize) reconstruct the sequence of inserts. For 281 × 3006 cotton containing three times the pUbi promoter, it was possible to propose contigs for each repetition of the promoter with its respective structural element (Fig. 3), which shows that the method is capable of proposing solutions to help to characterise complex sequences introduced into plants or even mixed GMOs. A sample containing 50% of A2704 soybean (construct: 35 S promoter – pat gene – 35 S terminator) and 50% of LL62 rice (construct: 35 S promoter – *bar* gene –35 S terminator) was also tested (Fig. 3). The bioinformatic pipeline was able to propose a sequence for the inserts introduced in each GM plant. The two sequences were clearly distinguishable even though the sequence of the *pat* and *bar* genes showed approximately 60% similarity when aligned. The sequences of the inserts introduced into A2704 soybean and LL62 rice are not publicly available. Therefore, no comparison between the obtained sequences with the announced sequences was possible. However, the percentage of similarity between known the *pat* and *bar* sequences falls into the same range.

Disruptions in the contigs were mainly due to the presence of structural elements that were not originally considered for enrichment and therefore constituted gaps, preventing reassembly of the whole sequence. Adding these elements to future enrichment steps would be an interesting recommendation. A definite advantage of this technology is that fragments caught by the capture probes covered junction regions as well, so it was not only possible to create contigs including junctions between structural elements but also between plant DNA and the GM construct (Figs 3 and 4).

The length of the contigs also depended on the fraction of the GMO in the analysed flour. An example is presented for GTS-40-3-2 soybean (Fig. 3), for which it was possible to assemble contigs even at a percentage as low as 0.1% of an event, proving that the methodology is very sensitive, as it still succeeded in characterising GMOs at low percentages. For GTS-40-3-2, at a level as low as 1% of GM, it was possible to recreate the transgenic construct and determine the left border (plant-GM construct junction) and the rearranged sequence as described by Windels *et al*.^28^ on the right side. This rearrangement corresponds to a portion of the EPSPS gene and a part of the plasmid vector used for transformation. The contig for GTS40-3-2 soybean at 1% was somewhat shorter than the contig obtained for GTS40-3-2 at 10%. At 0.1%, it was possible to create two contigs, with one of them covering the left junction (plant - DNA construct). The lower number of reads available in this last case made it impossible to reconstruct the whole sequence of the transgenic cassette.

DNA enrichment has a cost of 300 euros/sample and sequencing adds additional 300 euros/sample. This price is high for an analysis in the field of agrofood products, but since the first experiments, conducted 3 years ago, the estimated cost of the approach has already been halved. If the time required to perform enrichment (2 days), sequence the libraries (2 days) and complete the bioinformatics analysis (3 hours/sample) is reasonable for a routine analysis, access to a sequencing machine - if outsourced – generally takes at least one month and remains a very limiting factor when a fast answer is needed. Therefore, the use of affordable machines (e.g., minion, Oxford Nanopore technologies, Oxford, UK) must be tested in future approaches^29^.

The sequencing approach can be used: (i) alone, as a new detection and characterisation technique that has a good coverage because of the large number of structural elements tested; (ii) as a complement to real-time PCR to characterise the GM construct(s) or event(s) initially detected by real-time PCR tests; and (iii) prior to the development of an event-specific real-time PCR test because of the characterisation of the GM insert and its border regions.

Approaches to GMO detection using NGS have been proposed before, but this is the first time that such a methodology (i) enables the detection of GMOs at low levels, (ii) can be used on products containing several plant species, (iii) focuses on a large panel of screening elements, and (iv) makes it possible to partially or completely reconstruct a GMO, thereby providing a mechanism to detect unknown events. In the case of a laboratory equipped with NGS technology, this methodology could also be applied in a time frame that is more suitable for routine analysis.

Moreover, this is the first step towards a more informative analysis, as the enrichment can be extended to sequences corresponding to additional structural elements, plant species, allergens and contaminants. Specific sequences for these elements can be added to the database for the design of capture probes, leading to a technology not only focused on GMO detection but also extendable to the determination of other interesting food and feed product features. The strategy described in this study is only valid for GMOs obtained through classical recombinant DNA technology that give rise to transgene constructs. This study is not aimed at gene editing techniques (e.g., CRISPR/Cas9).

## Methods

### Samples

The certified transgenic reference materials (CRMs) were obtained from the Institute for Reference Materials and Measurements (JRC, Geel, Belgium) and the American Oil Chemists’ Society (AOCS, Urbana, Illinois, USA). Commercial organic grains were collected for non-GM plant species. Tests performed using real-time PCR^30,31^ confirmed the absence of GM material from commercial organic grains. The origin of the material is presented in the supplementary material (Table 2).

The samples were considered individually for sequencing (with the exception of the 50% LL62 rice/ 50% A2704-12 soybean mix), and some of the samples (maize 0% GM, soybean 0% GM, maize MON89034 100%) were repeated to observe background noise.

### DNA extraction

Genomic DNA was extracted and purified from all samples following the CTAB-based method described in Annex A.3.1 of the ISO 21571:2005 international standard^32^. The quality of DNA extracted from samples was estimated using a Nanodrop ND-1000 spectrophotometer (Nanodrop Technologies, Wilmington, DE). DNA samples were quantified by Picogreen (Quant-iT™ PicoGreen™ dsDNA Assay Kit, Invitrogen, Carlsbad, CA); 3 µg of DNA was used for library preparation.

### Next generation sequencing

DNA was sheared on a Picoruptor (Diagenode, Liège, Belgium) to produce fragments of ~150–200 bp. The SureSelect XT Target Enrichment system (Agilent technologies) was used to capture sequences of interest prior to sequencing. The design includes 458 enrichment probes. The sequences of the probes are available in supplementary material (Table S1). Via the online tool “Suredesign” on the Agilent Technologies website and through the option “collaboration space” with reference to design ID 3045501, probes were ordered from Agilent. No degeneracy was introduced in the sequences of the probes. Sequencing was performed on an Illumina MiSeq instrument with MiSeq Reagent Kit v3 (2 × 75 bp) at the GIGA Genomics platform at the University of Liège.

The pipeline for analysing results was perfected, as shown in Fig. 2, by the use of free access programs (with their default settings): bwa mem version 0.7.16-r1180^33^, Samtools version 0.1.19-96b5f2294a^34^, R ggplot version 2_2.2.1^35^, Seqtk version 1.2^36^, Velvet version 1.2.10^37^, Mira version 4.0.2^38^, Spades version 3.12^39^, CISA version 1.3^40^ and Blast version 2.7.1+^41,42^. The commands calling the different packages and an example (manifest file) are given in Supplementary Material S2.

The assembled contigs were compared to the sequences of the inserts introduced in plants: GTS 40-3-2 soybean (Windels *et al*., 2001), GT73 rapeseed (patent US 6248876), MS8 (structure of the plasmid pTHW101 as described in notification C/BE/96/01), 59122 maize (NCBI accession HW057200), 281 × 3006 cotton (patents EP2333082 and EP2862934) and MON89034 maize (NCBI accession FV532179).

## Supplementary information

Bioinformatic commands
