# Supplementary material for: Detection and identification of transgenic events by next generation sequencing combined with enrichment technologies
Source: Sci Rep. 2019 Oct 30;9:15595. doi: 10.1038/s41598-019-51668-x (PMC6821802; doi:10.1038/s41598-019-51668-x)
Supplement: Supplementary file 1 — Bioinformatic commands [file 41598_2019_51668_MOESM1_ESM.pdf]

Detection and identification of transgenic events by next generation sequencing combined with enrichment technologies

Frédéric Debode, Julie Hulin, Benoît Charloteaux, Wouter Coppieters, Marc Hanikenne, Latifa Karim and Gilbert Berben

Supplementary Table S1. Sequences of the probes used for enrichment

Structural element	Probe sequences
p35S	CGATGGCCTTTCCTTTATCGCAATGATGGCATTTGTAGGAGCCACCTTCCTTTTCCACTATCTTCACA ATAAAGTGACAGATAGCTGGGCAATGGAATCCGAGGAGGTTTCCGGATATTA
	CCCTTTGTTGAAAAGTCTCAATTGCCCTTTGGTCTTCTGAGACTGTATCTTTGATATTTTTGGAGTAG ACAAGTGTGTCGTGCTCCACCATGTTGACGAAGATTTTCTTCTTGTCATTGA
	TTTTGACTGCATCTTTAACCTTCTTGGGAAGGTATTTGATTTCCTGGAGATTATTGCTCGGGTAGATC GTCTTGATGAGACCTGCTGCGTAAGCCTCTCTAACCATCTGTGGGTTAGCAT
	TGGAGATATCACATCAATCCACTTGCTTTGAAGACGTGGTTGGAACGTCTTCTTTTTCCACGATGCTC CTCGTGGGTGGGGGTCCATCTTTGGGACCACTGTCGGCAGAGGCATCTTCAA
	TCTTGAAAGATCTGCTAGAGTCAGCTTGTCAGCGTGTTCTCTCCAAATGAAATGAACTTCCTTATATA GAGGAAGGGTCTTGCGAAGGATAGTGGGATTGTGCGTCATCCCTTACGTCAG
	GTCGTAAGAGACTCTGTATGAACTGTTCGCCAGTCTTTACGGCGAGTTCTGTTAGGTCCTCTATTTG AATCTTTGACTCCATGGCCTTTGATTCAGTGGGAACTACCTTTTTAGAGACTC
	CAATCTCTATTACTTGCCTTGGTTTGTGAAGCAAGCCTTGAATCGTCCATACTGGAATAGTACTTCTG ATCTTGAGAAATATATCTTTCTCTGTGTTCTTGATGCAGTTAGTCCTGAATC
	GAAGGTATTTGATTTCCTGGAGATTATTGCTCGGGTAGATCGTCTTGATGAGACCTGCTGCGTAAGC CTCTCTAACCATCTGTGGGTTAGCATTCTTTCTGAAATTGAAAAGGCTAATCT
pFMV	AATTCTCAGTCCAAAGCCTCAACAAGGTCAGGGTACAGAGTCTCCAAACCATTAGCCAAAAGCTACA GGAGATCAATGAAGAATCTTCAATCAAAGTAAACTACTGTTCCAGCACATGCA
	AACAAAATAACGTGGAAAAGAGCTGTCCTGACAGCCCACTCACTAATGCGTATGACGAACGCAGTG ACGACCACAAAAGAATTCCCTCTATATAAGAAGGCATTCATTCCCATTTGAAGG
	AAAGTAATCTTGTCAACATCGAGCAGCTGGCTTGTGGGGACCAGACAAAAAAGGAATGGTGCAGAA TTGTTAGGCGCACCTACCAAAAGCATCTTTGCCTTTATTGCAAAGATAAAGCAG
	ATTCCTCTAGTACAAGTGGGGAACAAAATAACGTGGAAAAGAGCTGTCCTGACAGCCCACTCACTAA TGCGTATGACGAACGCAGTGACGACCACAAAAGAATTCCCTCTATATAAGAAG
	TACAGGAGATCAATGAAGAATCTTCAATCAAAGTAAACTACTGTTCCAGCACATGCATCATGGTCAGT AAGTTTCAGAAAAAGACATCCACCGAAGACTTAAAGTTAGTGGGCATCTTTG
	AAATTGGTATCGCCAAAACCAAGAAGGAACTCCCATCCTCAAAGGTTTGTAAGGAAGAATTCTCAGT CCAAAGCCTCAACAAGGTCAGGGTACAGAGTCTCCAAACCATTAGCCAAAAGC

	GGTGCAGAATTGTTAGGCGCACCTACCAAAAGCATCTTTGCCTTTATTGCAAAGATAAAGCAGATTCCTCTAGTACAAGTGGGGAACAAAATAACGTGGAAAAGAGCTGTCCTGACAGCC
	TCATGGTCAGTAAGTTTCAGAAAAAGACATCCACCGAAGACTTAAAGTTAGTGGGCATCTTTGAAAGTAATCTTGTCAACATCGAGCAGCTGGCTTGTGGGGACCAGACAAAAAAGGAAT
	CACTCACTAATGCGTATGACGAACGCAGTGACGACCACAAAAGAATTAGCTTGAGCTCAGGATTTAGCAGCATTCCAGATTGGGTTCAATCAACAAGGTACGAGCCATATCACTTTATTC
pUbi	GCCCGGTAGTTCTACTTCTGTTCATGTTTGTGTTAGATCCGTGTTTGTGTTAGATCCGTGCTGCTAGCGTTCGTACACGGATGCGACCTGTACGTCAGACACGTTCTGATTGCTAACTTG
	TGGCATATGCAGCATCTATTCATATGCTCTAACCTTGAGTACCTATCTATTATAATAAACAAGTATGTTTTATAATTATTTTGATCTTGATATACTTGGATGATGGCATATGCAGCAGCT
	CCAGTGTTTCTCTTTGGGGAATCCTGGGATGGCTCTAGCCGTTCCGCAGACGGGATCGATTTCATGATTTTTTTTGTTTCGTTGCATAGGGTTTGGTTTGCCCTTTTCCTTTATTTCAAT
	AGTTTTTTTATTTAATAATTTAGATATAAAATAGAATAAAATAAAGTGACTAAAAATTAAACAAATACCCTTTAAGAAATTAAAAAAACTAAGGAAACATTTTTCTTGTTTCGAGTAGAT
	AATTTTGGAACTGTATGTGTGTGTCATACATCTTCATAGTTACGAGTTTAAGATGGATGGAAATATCGATCTAGGATAGGTATACATGTTGATGTGGGTTTTACTGATGCATATACATGA
	AATGCCAGCCTGTTAAACGCCGTCGACGAGTCTAACGGACACCAACCAGCGAACCAGCAGCGTCGCGTCGGGCCAAGCGAAGCAGACGGCACGGCATCTCTGTCGCTGCCTCTGGACCCC
	CCATTTTATTAGTACATCCATTTAGGGTTTAGGGTTAATGGTTTTTATAGACTAATTTTTTTAGTACATCTATTTTATTCTATTTTAGCCTCTAAATTAAGAAAACTAAAACTCTATTTT
	GATGATGGCATATGCAGCAGCTATATGTGGATTTTTTTAGCCCTGCCTTCATACGCTATTTATTTGCTTGGTACTGTTTCTTTTGTCGATGCTCACCCTGTTGTTTGGTGTTACTTCTGC
	CAGCTACGGGGGATTCCTTTCCCACCGCTCCTTCGCTTTCCCTTCCTCGCCCGCCGTAATAAATAGACACCCCCTCCACACCCTCTTTCCCCAACCTCGTGTTGTTCGGAGCGCACACAC
	GGTGGATTTATTAATTTTGGATCTGTATGTGTGTGCCATACATATTCATAGTTACGAATTGAAGATGATGGATGGAAATATCGATCTAGGATAGGTATACATGTTGATGCGGGTTTTACT
	ATTCACGTCCAAATGGGGGCCACTTGGCTGCAGTGCAGCGTGACCCGGTCGTGCCCCTCTCTAGAGATAATGAGCATTGCATGTCTAAGTTATAAAAAATTACCACATATTTTTTTTGTC
	GATGCATATACAGAGATGCTTTTTGTTCGCTTGGTTGTGATGATGTGGTGTGGTTGGGCGGTCGTTCATTCGTTCTAGATCGGAGTAGAATACTGTTTCAAACTACCTGGTGTATTTATT
	GTTAGACATGGTCTAAAGGACAATTGAGTATTTTGACAACAGGACTCTACAGTTTTATCTTTTTAGTGTGCATGTGTTCTCCTTTTTTTTTGCAAATAGCTTCACCTATATAATACTTCAT
	ACACTTGTTTGAAGTGCAGTTTATCTATCTTTATACATATATTTAAACTTTACTCTACGAATAATATAATCTATAGTACTACAATAATATCAGTGTTTTAGAGAATCATATAAATGAACA
	ATATGCCGTGCACTTGTTTGTCGGGTCATCTTTTCATGCTTTTTTTTGTCTTGGTTGTGATGATGTGGTCTGGTTGGGCGGTCGTTCTAGATCGGAGTAGAATTCTGTTTCAAACTACCT
	TCTCGAGAGTTCCGCTCCACCGTTGGACTTGCTCCGCTGTCGGCATCCAGAAATTGCGTGGCGGAGCGGCAGACGTGAGCCGGCACGGCAGGCGGCCTCCTCCTCCTCTCACGGCACCGG
	ACACAACCAGATCTCCCCCAAATCCACCCGTCGGCACCTCCGCTTCAAGGTACGCCGCTCGTCCTCCCCCCCCCCCCCTCTCTACCTTCTCTAGATCGGCGTTCCGGTCCATGGTTAGG

pNOS	AGAATTAAGGGAGTCACGTTATGACCCCCGCCGATGACGCGGGACAAGCCGTTTTACGTTTGGAACTGACAGAACCGCAACGATTGAAGGAGCCACTCAGCCGCGGGTTTCTGGAGTTTA
	ATGAGCTAAGCACATACGTCAGAAACCATTATTGCGCGTTCAAAAGTCGCCTAAGGTCACTATCAGCTAGCAAATATTTCTTGTCAAAAATGCTCCACTGACGTTCCATAAATTCCCCTC
	GATCCCGTCAATTCTCACTCATTAGGCACCCCAGGCTTTACACTTTATGCTTCCGGCTCGTATAATGTGTGGAATTGTGAGCGGATAACAATTTCACACAGGAAACAGGATCATGAGCGG
	TTCAAAAGTCGCCTAAGGTCACTATCAGCTAGCAAATATTTCTTGTCAAAAATGCTCCACTGACGTTCCATAAATTCCCCTCGGTATCCAATTAGAGTCTCATATTCACTCTCAATCAAA
pmas	AGTGTACCAAACAACGCTTTACAGCAAGAACGGAATGCGCGTGACGCTCGCGGTGACGCCATTTCGCCTTTTCAGAAATGGATAAATAGCCTTGCTTCCTATTATATCTTCCCAAATTAC
	TTTTCTTGTGGCCGTCTTTGTTTATATTGAGATTTTTCAAATCAGTGCGCAAGACGTGACGTAAGTATCCGAGTCAGTTTTTATTTTTCTACTAATTTGGTCGTTTATTTCGGCGTGTAG
	GGGCACCGGACAGGTCGGTCTTGACAAAAAGAACCGGGCGCCCCTGCGCTGACAGCCGGAACACGGCGGCATCAGAGCAGCCGATTGTCTGTTGTGCCCAGTCATAGCCGAATAGCCTCT
	ATTTCGCCTTTTCAGAAATGGATAAATAGCCTTGCTTCCTATTATATCTTCCCAAATTACCAATACATTACACTAGCATCTGAATTTCATAACCAATCTCGATACACCAAATCGATAAGC
	CCACCCAAGCGGCCGGAGAACCTGCGTGCAATCCATCTTGTTCAATCATAGATCCCCGTGTGGAAGATATGAATTTTTTTGAGAAACTAGATAAGATTAATGAATATCGGTGTTTTGGTT
	GACATGGCAACCGGGCCTGAATTTCGCGGGTATTCTGTTTCTATTCCAACTTTTTCTTGATCCGCAGCCATTAACGACTTTTGAATAGATACGCTGACACGCCAAGCCTCGCTAGTCAAA
Ps7s7	CTTTACAGCTATATCGATGTGACGTAAGATGCTTTACTTCGCTTCGAAGTAAAGTAGGAAATTGCTCGCTAAGTTATTCTTTTCTGAAAGAAATTAATTTAATTCTAATTAAATTAAATG
	ATTAGATGACACGTGGACGCTCAGGATCTGTGATGCTAGTGAAGCGCTTAAGCTGAACGAATCTGACGGAAGAGCGGACATACGCACATGGATTATGGCCCACATGTCTAAAGTGTATCT
	AATAGCCCTTGGATTAGATGACACGTGGACGCTCAGGATCTGTGATGCTAGTGAAGCGCTTAAGCTGAACGAATCTGACGGAAGAGCGGACATACGCACATGGATTATGGCCCACATGTC
	GAGACATCACGTGACTCTCACGTGATGTCTCCGCGACAGGCTGGCACGGGGCTTAGTATTACCCCCGTGCCGGGATCAGAGACATTTGACTAAATATTGACTTGGAATAATAGCCCTTGG
	TAATTAATAGTAATTATGATTAATTATGAGATAAGAGTTGTTATTAATGCTTATGAGGAATAAAGAATGATTAATATTGTTTAATTTTATTCCGCGAAGCGGTGTGTTATGTTTTTGTTG
	TAAAGTGTATCTCTTTACAGCTATATCGATGTGACGTAAGATGCTTTACTTCGCTTCGAAGTAAAGTAGGAAATTGCTCGCTAAGTTATTCTTTTCTGAAAGAAATTAATTTAATTCTAA
	ATGTTTTTGTTGGAGACATCACGTGACTCTCACGTGATGTCTCCGCGACAGGCTGGCACGGGGCTTAGTATTACCCCCGTGCCGGGATCAGAGACATTTGACTAAATATTGACTTGGAAT
	TATTCTTTTCTGAAAGAAATTAATTTAATTCTAAATTAAATTAAATGAGTGGCTATAAATAGTGTCGATGCTACCTCACATCGTATTCTTCTTCGCATCGTCTGTTCTGGTTTTAAGCGA
	AGTGGCCTGCAGTAATTAATAGTAATTATGATTAATTATGAGATAAGAGTTGTTATTAATGCTTATGAGGAATAAAGAATGATTAATATTGTTTAATTTTATTCCGCGAAGCGGTGTGTT
pRice_actin1	ATTTTTGAGAAAAATATATATTCAGGCGAATTCCACAATGAACAATAATAAGATTAAAATAGCTTGCCCCCGTTGCAGCGATGGGTATTTTTTCTAGTAAAATAAAAGATAAACTTAGAC

	ATTCGCGATTTGGAAATGCATATCTGTATTTGAGTCGGTTTTTAAGTTCGTTGCTTTTGTAAATACAGAGGGATTTGTATAAGAAATATCTTTAAAAAACCCATATGCTAATTTGACATA
	TAGCTAGCATACTCGAGGTCATTCATATGCTTGAGAAGAGAGTCGGGATAGTCCAAAATAAAACAAAGGTAAGATTACCTGGTCAAAAGTGAAAACATCAGTTAAAAGGTGGTATAAGTA
	TCAAAACATTTACAAAAACAACCCCTAAAGTCCTAAAGCCCAAAGTGCTATGCACGATCCATAGCAAGCCCAGCCCAACCCAACCCAACCCAACCCACCCCAGTGCAGCCAACTGGCAAA
	AAATATCGGTAATAAAAGGTGGCCCAAAGTGAAATTTACTCTTTTCTACTATTATAAAAATTGAGGATGTTTTGTCGGTACTTTGATACGTCATTTTTGTATGAATTGGTTTTTAAGTTT
	ATAGCAAGCCCAGCCCAACCCAACCCAACCCAACCCACCCCAGTGCAGCCAACTGGCAAATAGTCTCCACCCCCGGCACTATCACCGTGAGTTGTCCGCACCACCGCACGTCTCGCAGCC
pRice_actin2	CAGGTGGGTCCGGGTCGTGGGGGCCGGAAAAGCGAGGAGGATCGCGAGCAGCGACGAGGCCCGGCCCTCCCTCCGCTTCCAAAGAAACGCCCCCCATCGCCACTATATACATACCCCCCC
	ACCCTACCACCACCACCACCACCACCTCCTCCCCCCTCGCTGCCGGACGACGAGCTCCTCCCCCCTCCCCCTCCGCCGCCGCCGGTAACCACCCCGCCCCTCTCCTCTTTCTTTCTCCGT
	CTCTCCTCCCATCCCCCCAACCCTACCACCACCACCACCACCACCTCCTCCCCCCTCGCTGCCGGACGACGAGCTCCTCCCCCCTCCCCCTCCGCCGCCGCCGGTAACCACCCCGCCCCT
pSSuAra	TGCAAAAATACTGCTAGAGTAATGAAAAATATAATCTATTGCTGAAATTATCTCAGATGTTAAGATTTTCTTAAAGTAAATTCTTTCAAATTTTAGCTAAAAGTCTTGTAATAACTAAAG
	ATTTACTAGTAAAATAAATCACCAATTAGGTTTCTTATTATGTGCCAAATTCAATATAATTATAGAGGATATTTCAAATGAAAACGTATGAATGTTATTAGTAAATGGTCAGGTAAGACA
	GCTGGAATTTTTAATCTACATATTTGTTTTGGCCATGCACCAACTCATTGTTTAGTGTAATACTTTGATTTTGTCAAATATATGTGTTCGTGTATATTTGTATAAGAATTTCTTTGACCA
	TTAAAAAAATCCTACGTCAGATATTCAACTTTAAAAATTCGATCAGTGTGGAATTGTACAAAAATTTGGGATCTACTATATATATATAATGCTTTACAACACTTGGATTTTTTTTTGGAG
	TGCTAAATGCATAAGATTAGGTGAAACTGTGGAATATATATTTTTTTCATTTAAAAGCAAAATTTGCCTTTTACTAGAATTATAAATATAGAAAAATATATAACATTCAAATAAAAATGA
	TTGTTCTTCTTTACTCTTTGTGTGACTGAGGTTTGGTCTAGTGCTTTGGTCATCTATATATAATGATAACAACAATGAGAACAAGCTTTGGAGTGATCGGAGGGTCTAGGATACATGAGA
	CCTTTATGAACATTGAAAACTATACACAAAACAAGTCAGATAAATCTCTTTCTGGGCCTGTCTTCCCAACCTCCTACATCACTTCCCTATCGGATTGAATGTTTTACTTGTACCTTTTCC
	GTTGCAATGATATTGATAGTATGTTTGTGAAAACTAATAGGGTTAACAATCGAAGTCATGGAATATGGATTTGGTCCAAGATTTTCCGAGAGCTTTCTAGTAGAAAGCCCATCACCAGAA
	TTCAAGTGGACTAGGATCTACACCGTTGGATTTTGAGTGTGGATATGTGTGAGGTTAATTTTACTTGGTAACGGCCACAAAGGCCTAAGGAGAGGTGTTGAGACCCTTATCGGCTTGAAC
	CGCTGGAATAATGCCACGTGGAAGATAATTCCATGAATCTTATCGTTATCTATGAGTGAAATTGTGTGATGGTGGAGTGGTGCTTGCTCATTTTACTTGCCTGGTGGACTTGGCCCTTTC
	TATACACACACACATATATATATATATATATATATATTATATATCATGCACTTTTAATTGAAAAAATAATATATATATATATAGTGCATTTTTTCTAACAACCATATATGTTGCGATTGATC
	AAACTAAAGAGTCCACGAAAATATTACAAATCATAAGCCCAACAAAGTTATTGATCAAAAAAAAAAAACGCCCAACAAAGCTAAACAAAGTCCAAAAAAAACTTCTCAAGTCTCCATCTT

	CTTATGGGGAATTTATATTTTACTTACTATAGAGCTTTCATACCTTTTTTTTACCTTGGATTTAGTTAATATATAATGGTATGATTCATGAATAAAAATGGGAAATTTTTGAATTTGTAC
	TTATCTCAGATGTTAAGATTTTCTTAAAGTAAATTCTTTCAAATTTTAGCTAAAAGTCTTGTAATAACTAAAGAATAATACACAATCTCGACCACGGAAAAAAAACACATAATAAATTTG
	AAATAAGAACTTTCAAAAAACAGAACTATGTTTAATGTGTAAAGATTAGTCGCACATCAAGTCATCTGTTACAATATGTTACAACAAGTCATAAGCCCAACAAAGTTAGCACGTCTAAAT
pTA29	GAAGCTGTGCTAGAGAAGATGTTTATTCTAGTCCAGCCACCCACCTTATGCAAGTCTGCTTTTAGCTTGATTCAAAAACTGATTTAATTTACATTGCTAAATGTGCATACTTCGAGCCTA
	TGCAAATTATCCAGTTATACTTAGCTAGATATCCAATTTTGAATAAAAATAGCTCTTGATTAGTAAACCGGATAGTGACAAAGTCACATATCCATCAAACTTCTGGTGCTCGTGGCTAAG
	GATAGATTGAAAGAAGTGCAGGGAAGCGGTTAACTGGAACATAACACAATGTCTAAATTAATTGCATTCGCTAACCAAAAAGTGTATTACTCTCTCCGGTCCACAATAAGTTATTTTTTG
	GCAAAGTATCACAATTTTCTATCGAGAGATAGATTGAAAGAAGTGCAGGGAAGCGGTTAACTGGAACATAACACAATGTCTAAATTAATTGCATTCGCTAACCAAAAAGTGTATTACTCT
	TGTCGCTTTAATTCGAGTAGGATGTATATATTAGTACATAAAAAATCATGTTTGAATCATCTTTCATAAAGTGACAAGTCAATTGTCCCTTCTTGTTTGGCACTATATTCAATCTGTTAA
	TTCTGATCGACATGGGGTTAAAATTTAAATTGGGACACATAAATAGCCTATTTGTGCAAATCTCCCCATCGAAAATGACAGATTGTTACATGGAAAACAAAAAGTCCTCTGATAGAAGTC
pMTL	ACTAACGGCCGTCGTAAGAAGGTCCATCATGCGTGGAGCGAGCCCATTTGGTTGGTTGTCAGGCCGCAGTTAAGGCCTCCATATATGATTGTCGTCGGGCCCATAACAGCATCTCCTCCA
	CCAGTTTATTGTAAGAATAAATTAAGTAGAGATATTTGTCGTCGGGCAGAAGAAACTTGGACAAGAAGAAGAAGCAAGCTAGGCCAATTTCTTGCCGGCAAGAGGAAGATAGTGGCCTCT
	TCCATCAATTTAGAAATGAGCAAGCAAGCACCCGATCGCTCACCCCATATGCACCAATCTGACTCCCAAGCTCTGTTTCGCATTAGTACCGCCAGCACTCCACCTATAGCTACCAATTGA
	ATGCAATAATTCAGGGAATTCGTCAGCCAATTGAGGTAGCTAGTCAACTTGTACATTGGTGCGAGCAATTTTCCGCACTCAGGAGGGCTAGTTTGAGAGTCCAAAAACTATAGGAGATTA
	TGCTGGATTATTACTAAATCGAAATGGACGTAATATTCCAGGCAAGAATAATTGTTCGATCAGGAGACAAGTGGGGCATTGGACCGGTTCTTGCAAGCAAGAGCCTATGGCGTGGTGACA
	GGAGAGCGAGAGGCCAGAAATTTATAGCGACTGACGCTGTGGCAGGCACGCTATCGGAGGTTACGACGTGGCGGGTCACTCGACGCGGAGTTCACAGGTCCTATCCTTGCATCGCTCGGC
	TCCATCCTCACTTGCTATAAAAAGAGGTGTCCATGGTGCTCAAGCTCAGCCAAGCAAATAAGACGACTTGTTTCATTGATTCTTCAAGAGATCGAGCTTCTTTTGCACCACAAGGTCGAG
	AACAACACCCTCGCCATGCCGCTCGACTACAGCCTCCTCCTCGTCGGCGCCACAACGAGGGAGCCCGTGGTCGCAGCCACCGACCAGCATGTCTCTGTGTCCTCGTCCGACCTCGACATG
	CAAGCACGGCATGCATAGTATATGTATAGCTTGTTTTAGTGTGGCTTTGCTGAGACGAATGAAAGCAACGGCGGGCATATTTTTCAGTGGCTGTAGCTTTCAGGCTGAAAGAGACGTGGC
	GACCTTTCCAGCCTAAGCAGATCGATTGATCGTTAGAGTCAAAGAGTTGGTGGTACGGGTACTTTAACTACCATGGAATGATGGGGCGTGATGTAGAGCGGAAAGCGCCTCCCTACGCGG
	AAGAGGCTAAAATCCTCTCCTTATTTAATTTTAAATAAGTAGTGTATTTGTATTTTAACTCCTCCAACCCTTCCGATTTTATGGCTCTCAAACTAGCATTCAGTCTAATGCATGCATGCT

	TAAAGAAAATCGACGCAGGACAACCGTCAAAACGGGTGCTTTAATATAGTAGATATATATATATAGAG AGAGAGAGAAAGTACAAAGGATGCATTTGTGTCTGCATATGATCGGAGTATT
	TGGCTAGAGGTCGTATGGGGTTGTTAATAGCATAGCTAGCTACAAGTTAACCGGGTCTTTTATATTTA ATAAGGACAGGCAAAGTATTACTTACAAATAAAGAATAAAGCTAGGACGAAC
	CGCGGGAAAGCACGACGACTTACGAAGGTTAGTGGAGGAGGAGGACACACTAAAATCAGGACGCA AGAAACTCTTCTATTATAGTAGTAGAGAAGAGATTATAGGAGTGTGGGTTGATTC
	GATGTTATTTAAGAAAATATGTTGTTAACAGAATAAGATTAATATCGAAATGGAAACATCTGTAAATTA GAATCATCTTACAAGCTAAGAGATGTTCACGCTTTGAGAAACTTCTTCAGA
	TCATGACCGTAGAAGTAGCTCTCCAAGACTCAACGAAGGCTGCTGCAATTCCACAAATGCATGACAT GCATCCTTGTAACCGTCGTCGCCGCTATAAACACGGATAACTCAATTCCCTGC
	CGGCGCGTTGCCCATACATCATGCCTCCATCGATGATCCATCCTCACTTGCTATAAAAAGAGGTGTC CATGGTGCTCAAGCTCAGCCAAGCAAATAAGACGACTTGTTTCATTGATTCTT
	TCATGGCAAACAGTCGGACGCCAGCACCAGACTGACGACATGAGTCTCTGAAGAGCCCGCCACCTA GAAAGATCCGAGCCCTGCTGCTGGTAGTGGTAACCATTTTCGTCGCGCTGACGC
	AGTTTATATATCGGCGTGATGATGATGCTCCTAGCTAGAAATGAGAGAAGAAAAACGGACGCGTGTT TGGTGTGTGTCAATGGCGTCCATCCTTCCATCAGATCAGAACGATGAAAAAGT
	TATTCTATTTTTCATGATGTATCTTCCTCTAACATGCCTTAATTTGCAAATTTGGTATAACTACTGATTG AAAATATATGTATGTAAAAAAATACTAAGCATATTTTTGAAGCTAAACAT
	GCGGAGTTTACGGGGACTTATCCTTACGACGTGCTCTAAGGTTGCGATAACGGGCGGAGGAAGGC GTGTGGCGTGCGGAGACGGTTTATACACGTAGTGTGCGGGAGTGTGTTTCGTAGA
	TGCACATGACAACAATTGTAAGAGGATGGAGACCACAACGATCCAACAATACTTCTGCGACGGGCT GTGAAGTATAGAGAAGTTAAACGCCCAAAAGCCATTGTGTTTGGAATTTTTAGT
tOCS	GGATGATCCTCCAGCGCGGGGATCTCATGCTGGAGTTCTTCGCCCACCCCCTGCTTTAATGAGATA TGCGAGACGCCTATGATCGCATGATATTTGCTTTCAATTCTGTTGTGCACGTTG
	ATCCAATTTTAAAAAAAGCGGCAGAACCGGTCAAACCTAAAAGACTGATTACATAAATCTTATTCAAA TTTCAAAAGGCCCCAGGGGCTAGTATCTACGACACACCGAGCGGCGAACTAA
	GTACCCTACTACTTATATGTACAATATTAAAATGAAAACAATATATTGTGCTGAATAGGTTTATAGCGA CATCTATGATAGAGCGCCACAATAACAAACAATTGCGTTTTATTATTACAA
	TAACGTTCACTGAAGGGAACTCCGGTTCCCCGCCGGCGCGCATGGGTGAGATTCCTTGAAGTTGAG TATTGGCCGTCCGCTCTACCGAAAGTTACGGGCACCATTCAACCCGGTCCAGCA
	TTCAACCCGGTCCAGCACGGCGGCCGGGTAACCGACTTGCTGCCCCGAGAATTATGCAGCATTTTT TTGGTGTATGTGGGCCCTGTACAGCGGCCGCGTTAACGCGTATACTCTAGAGCG
	TAAAAAACCTGAGCATGTGTAGCTCAGATCCTTACCGCCGGTTTCGGTTCATTCTAATGAATATATCA CCCGTTACTATCGTATTTTTATGAATAATATTCTCCGTTCAATTTACTGATT
	GGACTCTGGGGTTCGAAATGACCGACCAAGCGACGCCCAACCTGCCATCACGAGATTTCGATTCCA CCGCCGCCTTCTATGAAAGGTTGGGCTTCGGAATCGTTTTCCGGGACGCCGGCT
tE9	TTGTACCATTTGTTGTGCTTGTAATTTACTGTGTTTTTTATTCGGTTTTCGCTATCGAACTGTGAAATG GAAATGGATGGAGAAGAGTTAATGAATGATATGGTCCTTTTGTTCATTCTC
	AAATTAATATTATTTGTTTTTTCTCTTATTTGTTGTGTGTTGAATTTGAAATTATAAGAGATATGCAAAC ATTTTGTTTTGAGTAAAAATGTGTCAAATCGTGGCCTCTAATGACCGAAG

	TTTAGACATTTATGGACTTTCCTTTATGTAATTTTCCAGAATCCTTGTCAGATTCTAATCATTGCTTTATAATTATAGTTATACTCATGGATTTGTAGTTGAGTATGAAAATATTTTTTA
	TTAATATGAGGAGTAAAACACTTGTAGTTGTACCATTATGCTTATTCACTAGGCAACAAATATATTTTCAGACCTAGAAAAGCTGCAAATGTTACTGAATACAAGTATGTCCTCTTGTGT
	CAGATTCTAATCATTGCTTTATAATTATAGTTATACTCATGGATTTGTAGTTGAGTATGAAAATATTTTTTAATGCATTTTATGACTTGCCAATTGATTGACAACATGCATCAATCGACC
	AGCTTTCGTTCGTATCATCGGTTTCGACAACGTTCGTCAAGTTCAATGCATCAGTTTCATTGCGCACACACCAGAATCCTACTGAGTTTGAGTATTATGGCATTGGGAAAACTGTTTTTC
tNOS	GTATTAAATGTATAATTGCGGGACTCTAATCATAAAAACCCATCTCATAAATAACGTCATGCATTACATGTTAATTATTACATGCTTAACGTAATTCAACAGAAATTATATGATAATCAT
	TCGACCGCGGTACCCGGAATTCGAGCTCGGTACCCGGGGATCTTCCCGATCTAGTAACATAGATGACACCGCGCGCGATAATTTATCCTAGTTTGCGCGCTATATTTTGTTTTCTATCGC
	GCATTACATGTTAATTATTACATGCTTAACGTAATTCAACAGAAATTATATGATAATCATCGCAAGACCGGCAACAGGATTCAATTTTAAGAAACTTTATTGCCAAATGTTTGAACGATC
tg7	TCTTGAAAGAAATATAGTTTAAATATTTATTGATAAAATAACAAGTCAGGTATTATAGTCCAAGCAAAAACATAAATTTATTGATGCAAGTTTAAATTCAGAAATATTTCAATAACTGAT
	ATGCAAGTTTAAATTCAGAAATATTTCAATAACTGATTATATCAGCTGGTACATTGCCGTAGATGAAAGACTGAGTGCGATATTATGTGTAATACATAAATTGATGATATAGCTAGCTTA
tpinII	AGACTTGTCCATCTTCTGGATTGGCCAACTTAATTAATGTATGAAATAAAAGGATGCACACATAGTGACATGCTAATCACTATAATGTGGGCATCAAAGTTGTGTGTTATGTGTAATTAC
	GATGAACCAGATGCATTTCATTAACCAAATCCATATACATATAAATATTAATCATATATAATTAATATCAATTGGGTTAGCAAAACAAATCTAGTCTAGGTGTGTTTTGCGAATTGCGGC
	TAGTTATCTGAATAAAAGAGAAAGAGATCATCCATATTTCTTATCCTAAATGAATGTCACGTGTCTTTATAATTCTTTGATGAACCAGATGCATTTCATTAACCAAATCCATATACATAT
t35S	TCACTCTCGGCATGGACGAGCTGTACAAGTGAAGATCCACCTAGTCTAGAGTCCGCAAAAATCACCAGTCTCTCTCTACAAATCTATCTCTCTCTATTTTTCTCCAGAATAATGTGTGAG
	CTCTATTTTTCTCCAGAATAATGTGTGAGTAGTTCCCAGATAAGGGAATTAGGGTTCTTATAGGGTTTCGCTCATGTGTTGAGCATATAAGAAACCCTTAGTATGTATTTGTATTTGTAA
gus	AGCCAGACAGAGTGTGATATCTACCCGCTTCGCGTCGGCATCCGGTCAGTGGCAGTGAAGGGCGAACAGTTCCTGATTAACCACAAACCGTTCTACTTTACTGGCTTTGGTCGTCATGAA
	GGAATGGTGATTACCGACGAAAACGGCAAGAAAAAGCAGTCTTACTTCCATGATTTCTTTAACTATGCCGGGATCCATCGCAGCGTAATGCTCTACACCACGCCGAACACCTGGGTGGAC
	ACTGGACAAGGCACTAGCGGGACTTTGCAAGTGGTGAATCCGCACCTCTGGCAACCGGGTGAAGGTTATCTCTATGAACTGTGCGTCACAGCCAAAAGCCAGACAGAGTGTGATATCTAC
	GAGCATCAGGGCGGCTATACGCCATTTGAAGCCGATGTCACGCCGTATGTTATTGCCGGGAAAAGTGTACGTATCACCGTTTGTGTGAACAACGAACTGAACTGGCAGACTATCCCGCCG
	GATATCACCGTGGTGACGCATGTCGCGCAAGACTGTAACCACGCGTCTGTTGACTGGCAGGTGGTGGCCAATGGTGATGTCAGCGTTGAACTGCGTGATGCGGATCAACAGGTGGTTGCA
gox	ATGGCTGAGAACCACAAGAAGGTTGGTATCGCTGGAGCTGGAATCGTTGGTGTTTGCACTGCTTTGATGCTTCAACGTCGTGGATTCAAGGTTACCTTGATTGATCCAAACCCACCAGGT

	ATGACCGCAACCCTCGTTTCTGAGCTCCTCGCAGGTGAGAAGACCTCTATCGACATCTCTCCATTCGCACCAAACCGTTTCGGTATTGGTAAGTCCAAGCAAACTGGTCCTGCATCCTAA
	ATCCCAGATTCCCTTCCAGTGATTGGTCGTGCTACCCGTACTCCAGACGTTATCTACGCTTTCGGTCACGGTCACCTCGGTATGACTGGTGCTCCAATGACCGCAACCCTCGTTTCTGAG
	ATCGCCAACCCAGAAGCTGCTCCACGTATTCCAACTACCGATGCTTCTGGAAAGTTCATCGCTACTCCTATGGAGATGGGTCTTCGTGTTGCTGGAACCGTTGAGTTCGCTGGTCTCACT
	CCTTTGATCAAGTCCTTGGCTGAGGAGGCTGATGCTAGCCACCTTATCCGTCACGAAGGTCACCTTACCGTGTACCGTGGAGAAGCAGACTTCGCCAAGGACCGTGGAGGTTGGGAACTT
	ACCAACGGTGTTCTTGCTGTTGATGCAGCTGTTGTTGCAGCTGGTGCACACTCCAAGTCTCTTGCTAACTCCCTTGGTGATGACATCCCATTGGATACCGAACGTGGATACCACATCGTG
	CGTCGTCTCAACGGTGTTCGTACTCAAATCCTCAGCGCTGATGCATTGCGTGATTTCGATCCTAACTTGTCTCACGCCTTTACCAAGGGAATCCTTATCGAAGAGAACGGTCACACCATC
	ATCCGTTTCAGCTACTTTCCAACCATCATGCCTTGGTTGATTCGTTTCTTGCTTGCTGGAAGACCAAACAAGGTGAAGGAGCAAGCTAAGGCACTCCGTAACCTCATCAAGTCCACTGTG
	GAAGGTGCTTCTTTCGGTAACGCTGGTTGCTTCAACGGTTCCTCCGTTGTTCCAATGTCCATGCCAGGAAACTTGACTAGCGTTCCAAAGTGGCTTCTTGACCCAATGGGTCCATTGTCC
	AACCCACAAGGTCTCGTGACTCTCTTGTTTCGTCGTTTCATCGCTAACGGTGGAGAGTTCGTGTCTGCTCGTGTTATCGGATTCGAGACTGAAGGTCGTGCTCTCAAGGGTATCACCACC
	GCTGCTCCTAACTGGAAGCGTGCTCACGTTCTCTACACTCACGCTCGTAAGTTGCTTCCAGCTCTCGCTCCTGCCAGTTCTGAAGAACGTTACTCCAAGTGGATGGGTTTCCGTCCAAGC
cryIAb_1	GACCCCACCAACCCAGCCCTGCGCGAGGAGATGCGCATCCAGTTCAACGACATGAACTCTGCCCTGACCACCGCCATCCCACTCTTCGCTGTCCAGAACTACCAGGTCCCTCTCCTGTCT
	ATCGAACAGTTAATTAACCAAAGAATAGAAGAATTCGCTAGGAACCAAGCCATCTCTAGACTGGAGGGCCTGAGCAACCTGTACCAGATCTACGCCGAGAGCTTCCGCGAGTGGGAGGCT
	AGCCAACGCTACAGGGTCAGGATCAGGTACGCTTCCACCACCAACCTGCAGTTCCACACCAGCATCGACGGCAGGCCCATCAACCAGGGCAACTTCAGCGCCACCATGAGCAGCGGCAGC
	TTCAGCAACAGCAGCGTGAGCATCATCAGGGCTCCCATGTTCAGCTGGATCCACCGCAGCGCTGAGTTCAACAACATCATTCCAAGTAGCCAGATCACTCAGATCCCACTCACCAAGAGC
	ATGGCTGACAACAACCCCAACATCAACGAGTGCATCCCCTACAACTGCCTGAGCAACCCAGAGGTGGAGGTGCTGGGTGGTGAGAGGATCGAGACCGGTTACACCCCCATCGACATCAGC
	GTATACCGCAAGAGCGGCACTGTGGACAGCCTGGACGAGATCCCACCCCAGAACAACAACGTGCCCCCTAGGCAGGGGTTCTCTCATCGCCTCTCACACGTGAGCATGTTCCGCAGCGGC
	TCCAACGGCAGCAGCGTGTTCACCCTGTCTGCCCACGTGTTCAACAGCGGCAACGAGGTGTACATCGACAGGATCGAGTTTGTCCCAGCTGAGGTGACCTTCGAAGCTGAGTACGACTGA
	CTGAGCCTGACCCAGTTCCTGCTGAGCGAGTTCGTGCCTGGTGCTGGCTTCGTGCTGGGACTAGTGGACATCATCTGGGGCATCTTCGGTCCCAGCCAGTGGGATGCCTTCCTGGTGCAG
	TTCAGGGGCTCTGCCCAGGGCATCGAGGGCAGCATCCGCAGCCCCCACCTGATGGACATCCTGAACAGCATCACCATCTACACTGACGCCCACAGGGGTGAGTACTACTGGTCTGGCCAC
	CGCACCCTGAGCAGCACCCTGTACAGGAGGCCCTTCAACATCGGCATCAACAACCAGCAGCTGAGCGTGCTGGATGGCACCGAGTTCGCCTACGGCACCAGCAGCAACCTGCCCAGCGCC

	CAGATCATGGCTTCTCCCGTGGGCTTCAGCGGTCCCGAGTTCACCTTCCCCCTGTACGGCACAATG GGCAACGCTGCCCCACAGCAGAGGATCGTGGCCCAGCTGGGCCAGGGCGTGTAC
	GTCTTGGATATCGTCAGTCTCTTCCCCAACTACGACAGCAGGACCTACCCTATCCGGACTGTGAGC CAGCTGACCCGCGAGATCTACACCAACCCCGTGCTGGAGAACTTCGACGGCAGC
	AACCTGCAGAGCGGAAGCTTCCGCACTGTGGGCTTCACTACCCCATTCAACTTCTCCAACGGCAGC AGCGTGTTCACCCTGTCTGCCCACGTGTTCAACAGCGGCAACGAGGTGTACATC
	ACCAACCTGGGCTCCGGGACTAGCGTTGTCAAGGGACCAGGGTTCACTGGAGGCGACATCCTGAG GAGGACCAGCCCAGGCCAGATCAGCACCTTAAGGGTGAACATCACCGCTCCCCTC
	ATCGGCAACTACACCGACCACGCAGTGAGATGGTACAACACTGGGCTTGAGAGGGTCTGGGGTCC CGACAGCCGCGACTGGATCAGGTACAACCAGTTCAGGCGTGAACTCACTCTCACC
	GTCTATGTGCAAGCTGCCAACCTCCATCTCAGCGTCCTTCGCGACGTGAGCGTCTTTGGGCAGAGG TGGGGGTTCGACGCTGCCACCATCAACAGCCGCTACAACGACCTGACGCGTCTG
crylab_2	TGGATTCATCGCAGCGCGGAGTTCAACAATATCATTCCGTCCTCCCAAATCACCCAAATCCCCCTCA CCAAGTCCACCAACCTGGGCAGCGGCACCTCCGTGGTGAAGGGCCCAGGCTTC
	CACCTGATGGACATCCTCAACAGCATCACTATCTACACCGATGCCCACCGCGGCGAGTACTACTGG TCCGGCCACCAGATCATGGCCTCCCCGGTCGGCTTCAGCGGCCCCGAGTTTACC
	CGGTACGCGCTCACTCCGCCCTCTGCCTTTGTTACTGCCACGTTTCTCTGAATGCTCTCTTGTGTGG TGATTGCTGAGAGTGGTTTAGCTGGATCTAGAATTACACTCTGAAATCGTGTT
	TTCAACTTCTCCAACGGCTCCAGCGTTTTCACCCTGAGCGCCCACGTGTTCAATTCCGGCAATGAGG TGTACATTGACCGCATTGAGTTCGTGCCAGCCGAGGTCACCTTCGAAGCCGAG
	CAGGCCATCAGCCGCCTGGAGGGCCTCAGCAACCTCTACCAAATCTACGCTGAGAGCTTCCGCGA GTGGGAGGCCGACCCCACTAACCCAGCTCTCCGCGAGGAGATGCGCATCCAGTTC
	AACGACATGAACAGCGCCCTGACCACCGCCATCCCACTCTTCGCCGTCCAGAACTACCAAGTCCCG CTCCTGTCCGTGTACGTCCAGGCCGCCAACCTGCACCTCAGCGTGCTGAGGGAC
	TCATCATGAGAATCCTTCTTAGATGTAAGACCACCTGCAGCAGATGACTTTGATCTTGTTGTTGGGC GCCGACCAGATTGAGCCATTGCAGCTGTTAATGATGCACCAGCCGTGGTGCCA
	TTGATGTTTATCTCTGCTCCTTTATTGTGACCATAAGTCAAGATCAGATGCACTTGTTTTAAATATTGT TGTCTGAAGAAATAAGTACTGACAGTATTTTGATGCATTGATCTGCTTGTT
	ATCCTACTAGACTGGCTGAGCACAACAGGGAAACGTTCCAGCGAAGACAATGCACTTTGCAGTTTCT GAACCAACAGTGCCATGGGAGTTTCATCTCTCTCGACCAATCGAATTTCCGCG
	ATAATAATTTGTTCGTCTGCAGAGCTTATTATTTGCCAAAATTAGATATTCCTATTCTGTTTTTGTTTGT GTGCTGTTAAATTGTTAACGCCTGAAGGAATAAATATAAATGACGAAATT
	GAGGACTTTCGGTAGCCTTCTTTCATTTCCGAATTTGCTTGCGAGCAGTCAGGTCCTTTTGATTCATC TGAGTTTGGCTTTACAATAGCTTTTCCTTTTCCTTTGGCAGTACTAGTGCTT
	ACGGGCGGCGACATCCTGCGCAGGACCTCCCCGGGCCAGATCAGCACCCTCCGCGTCAACATCAC CGCTCCCCTGTCCCAGAGGTACCGCGTCAGGATTCGCTACGCTAGCACCACCAAC
	TACGACCTGGAGAGAGCCCAGAAGGCTGTCAATGAGCTCTTCACGTCCAGCAATCAGATCGGCCTG AAGACCGACGTCACTGACTACCACATCGACCAAGTCTCCAACCTCGTGGAGTGC
	TTTCCTCTCTACGGCACGATGGGCAACGCCGCTCCACAACAACGCATCGTCGCTCAGCTGGGCCAG GGCGTCTACCGCACCCTGAGCTCCACCCTGTACCGCAGGCCCTTCAACATCGGT

	CTGGAGCGCGTCTGGGGCCCTGATTCTAGAGACTGGATTCGCTACAACCAGTTCAGGCGCGAGCT<br>GACCCTCACCGTCCTGGACATTGTGTCCCTCTTCCCGAACTACGACTCCCGCACC
	TGCCTCAGCAACCCTGAGGTCGAGGTGCTCGGCGGTGAGCGCATCGAGACCGGTTACACCCCCAT<br>CGACATCTCCCTCTCCCTCACGCAGTTCCTGCTCAGCGAGTTCGTGCCAGGCGCT
	CAGCTCGACCGCGGCTGGCGCGGCAGCACCGACATCACGATCCAGGGCGGCGACGATGTGTTCAA<br>GGAGAACTACGTGACTCTCCTGGGCACTTTCGACGAGTGCTACCCTACCTACTTG
	TACCAGAAGATCGATGAGTCCAAGCTCAAGGCTTACACTCGCTACCAGCTCCGCGGCTACATCGAA<br>GACAGCCAAGACCTCGAGATTTACCTGATCCGCTACAACGCCAAGCACGAGACC
	GTCAGCGTGTTTGGCCAGAGGTGGGGCTTCGACGCCGCCACCATCAACAGCCGCTACAACGACCT<br>CACCAGGCTGATCGGCAACTACACCGACCACGCTGTCCGCTGGTACAACACTGGC
	TGTTGTAACAAAATTTAAAAATAAAGAGTTTCCTTTTTGTTGCTCTCCTTACCTCCTGATGGTATCTAG<br>TATCTACCAACTGACACTATATTGCTTCTCTTTACATACGTATCTTGCTCG
	ATCAACAACCAGCAGCTGTCCGTCCTGGATGGCACTGAGTTCGCCTACGGCACCTCCTCCAACCTG<br>CCCTCCGCTGTCTACCGCAAGAGCGGCACGGTGGATTCCCTGGACGAGATCCCA
	GTCAACGTGCCCGGTACTGGTTCCCTCTGGCCGCTGAGCGCCCCCAGCCCGATCGGCAAGTGTGC<br>CCACCACAGCCACCACTTCTCCTTGGACATCGATGTGGGCTGCACCGACCTGAAC
	TACCCGATCCGCACCGTGTCCCAACTGACCCGCGAAATCTACACCAACCCCGTCCTGGAGAACTTC<br>GACGGTAGCTTCAGGGGCAGCGCCCAGGGCATCGAGGGCTCCATCAGGAGCCCA
	CTGCAATTCCACACCTCCATCGACGGCAGGCCGATCAATCAGGGTAACTTCTCCGCCACCATGTCC<br>AGCGGCAGCAACCTCCAATCCGGCAGCTTCCGCACCGTGGGTTTCACCACCCCC
	CTGCCTGTGCTGATTACTTGCCGTCCTTTGTAGCAGCAAAATATAGGGACATGGTAGTACGAAACGA<br>AGATAGAACCTACACAGCAATACGAGAAATGTGTAATTTGGTGCTTAGCGGTA
	TACTAGACTGGCTGAGCACAACAGGGAAACGTTCCAGCGAAGACAATGCACTTTGCAGTTTCTGAAC<br>CAACAGTGCCATGGGAGTTTCATCTCTCTCGACCAATCGAATTTCCGCGGGCC
	TTTATTTAAGCACATGTTGGTGTTATAGGGCACTTGGATTCAGAAGTTTGCTGTTAATTTAGGCACAG<br>GCTTCATACTACATGGGTCAATAGTATAGGGATTCATATTATAGGCGATACT
	ATGCCTTCTCCCTAGTGTTGACCAGTGTTACTCACATAGTCTTTGCTCATTTCATTGTAATGCAGATA<br>CCAAGCGGCCATGGACAACAACCCAAACATCAACGAGTGCATCCCGTACAAC
	CTCTCCGATGAGTTCTGCCTCGACGAGAAGAAGGAGCTGTCCGAGAAGGTGAAGCATGCCAAGCGT<br>CTCAGCGACGAGAGGAATCTCCTCCAGGACCCCAATTTCCGCGGCATCAACAGG
	CCACAGAACAACAATGTGCCCCCCAGGCAGGGTTTTTCCCACAGGCTCAGCCACGTGTCCATGTTC<br>CGCTCCGGCTTCAGCAACTCGTCCGTGAGCATCATCAGAGCTCCTATGTTCTCC
	GGCTTCGTCCTGGGCCTCGTGGACATCATCTGGGGCATCTTTGGCCCCTCCCAGTGGGACGCCTTC<br>CTGGTGCAAATCGAGCAGCTCATCAACCAGAGGATCGAGGAGTTCGCCAGGAAC
	AGCACAATATTTGATGAATAATCCCGAAGTGATTTTTCGCCATGAGCTCGGGAAAGACGAAGCTTGA<br>AGGGTTGAGCCAGAGCACTAAGACCTGAAGTCAGACGAGACCCTCCAATACCA
	GGAACCCCAGATTCAGAATTATTACCAGATGGAATTATAGGCTTCGATGCAACCTCACTGCGTTGAA<br>CTCTAGGCCAAAGGAATTCTTCAACAGATGCAAGACTAGCAAATGGGTCGATA
cryIAb_3	GGCAACTACACCGACCACGCCGTGCGCTGGTACAACACCGGCCTGGAGCGCGTGTGGGGTCCCG<br>ACAGCCGCGACTGGATCAGGTACAACCAGTTCCGCCGCGAGCTGACCCTGACCGTG

	CGCGGCAGCGCCCAGGGCATCGAGGGCAGCATCCGCAGCCCCCACCTGATGGACATCCTGAACA<br>GCATCACCATCTACACCGACGCCCACCGCGGCGAGTACTACTGGAGCGGCCACCAG
	CTGGACATCGTGAGCCTGTTCCCCAACTACGACAGCCGCACCTACCCCATCCGCACCGTGAGCCAG<br>CTGACCCGCGAGATTTACACCAACCCCGTGCTGGAGAACTTCGACGGCAGCTTC
	AACCTGGGCAGCGGCACCAGCGTGGTGAAGGGCCCCGGCTTCACCGGCGGCGACATCCTGCGCC<br>GCACCAGCCCCGGCCAGATCAGCACCCTGCGCGTGAACATCACCGCCCCCCTGAGC
	AGCAACAGCAGCGTGAGCATCATCCGTGCACCTATGTTCAGCTGGATTCACCGCAGTGCCGAGTTC<br>AACAACATCATCCCCAGCAGCCAAATCACCCAGATCCCCCTGACCAAGAGCACC
	GAGCAGCTGATCAACCAGCGCATCGAGGAGTTCGCCCGCAACCAGGCCATCAGCCGCCTGGAGGG<br>CCTGAGCAACCTGTACCAAATCTACGCCGAGAGCTTCCGCGAGTGGGAGGCCGAC
	CTGCAGAGCGGCAGCTTCCGCACCGTGGGCTTCACCACCCCCTTCAACTTCAGCAACGGCAGCAG<br>CGTGTTCACCCTGAGCGCCCACGTGTTCAACAGCGGCAACGAGGTGTACATCGAC
	TACCGCAAGAGCGGCACCGTGGACAGCCTGGACGAGATCCCCCCTCAGAACAACAACGTGCCACC<br>TCGACAGGGCTTCAGCCACCGTCTGAGCCACGTGAGCATGTTCCGCAGTGGCTTC
	TACGTGCAGGCCGCCAACCTGCACCTGAGCGTGCTGCGCGACGTCAGCGTGTTCGGCCAGCGCTG<br>GGGCTTCGACGCCGCCACCATCAACAGCCGCTACAACGACCTGACCCGCCTGATC
	CGCATCGAGTTCGTGCCCGCCGAGGTGACCTTCGAGGCCGAGTACGACCTGGAGAGGGCTCAGAA<br>GGCCGTGAACGAGCTGTTCACCAGCAGCAACCAGATCGGCCTGAAGACCGACGTG
	CAGCGCTACCGCGTCCGCATCCGCTACGCCAGCACCACCAACCTGCAGTTCCACACCAGCATCGA<br>CGGCCGCCCCATCAACCAGGGCAACTTCAGCGCCACCATGAGCAGCGGCAGCAAC
	ACCCTGAGCAGCACCCTGTACCGTCGACCTTTCAACATCGGCATCAACAACCAGCAGCTGAGCGTG<br>CTGGACGGCACCGAGTTCGCCTACGGCACCAGCAGCAACCTGCCCAGCGCCGTG
	ACCTTCGAGGCCGAGTACGACCTGGAGAGGGCTCAGAAGGCCGTGAACGAGCTGTTCACCAGCAG<br>CAACCAGATCGGCCTGAAGACCGACGTGACCGACTACCACATCGATCAGGTGTAG
	ATGGACAACAACCCCAACATCAACGAGTGCATCCCCTACAACTGCCTGAGCAACCCCGAGGTGGAG<br>GTGCTGGGCGGCGAGCGCATCGAGACCGGCTACACCCCCATCGACATCAGCCTG
	CCCACCAACCCCGCCCTGCGCGAGGAGATGCGCATCCAGTTCAACGACATGAACAGCGCCCTGAC<br>CACCGCCATCCCCCTGTTCGCCGTGCAGAACTACCAGGTGCCCCTGCTGAGCGTG
	AGCCTGACCCAGTTCCTGCTGAGCGAGTTCGTGCCCGGCGCCGGCTTCGTGCTGGGCCTGGTGGA<br>CATCATCTGGGGCATCTTCGGCCCCAGCCAGTGGGACGCCTTCCTGGTGCAGATC
	ATCATGGCCAGCCCCGTCGGCTTCAGCGGCCCCGAGTTCACCTTCCCCCTGTACGGCACCATGGG<br>CAACGCTGCACCTCAGCAGCGCATCGTGGCACAGCTGGGCCAGGGAGTGTACCGC
cry1B	GTGTACTCTTGGACTCACAGGTCTGCTGACCGCACTAACACCATCGGCCCAAACAGGATCACCCAA<br>ATCCCAATGGTGAAGGCCTCCGAGCTGCCACAAGGCACCACCGTGGTGAGGGGA
	GCCCAGTTGACCAGAGAGGTTTACACCGATGCTATCGGTGCTACTGGCGTGAACATGGCCAGCATG<br>AACTGGTACAACAACAACGCCCCATCCTTCTCCGCCATCGAAGCTGCCGCTATC
	ATGGTTACCTCCAACCGTAAGAACGAGAACGAGATCATCAACGCTGTGTCCAACCACTCCGCTCAG<br>ATGGACCTCCTCCCAGACGCTCGCATCGAGGACTCCTTGTGCATCGCTGAGGGC
	TTCCTCAACGCTATGCCACTCTTCGCCATCAGGAACCAAGAGGTCCCATTGTTGATGGTGTACGCTC<br>AGGCTGCCAACTTGCACCTCTTGTTGTTGAGGGACGCCTCCCTCTTCGGCAGC

	AGGTCCCCACACCTCCTGGACTTCCTGGAGCAACTCACCATCTTCAGCGCTTCCTCCCGCTGGAGC<br>AACACCAGGCACATGACCTACTGGCGTGGTCACACCATCCAGTCCCGTCCCATC
	CCAGGCTTCACTGGCGGCGACATCCTCAGGAGGACCAACACTGGCGGCTTCGGTCCCATCAGGGT<br>CACCGTCAACGGCCCATTGACCCAAAGATACCGCATCGGCTTCCGCTACGCTTCC
	GGAGGTGGCTTGAACACCTCCACCCACGGAGCTACTAACACCTCCATCAACCCAGTGACCTTGCGT<br>TTCGCCTCTCGTGACGTGTACAGGACCGAGAGCTACGCCGGTGTCCTCCTCTGG
	AACAACATCGACCCATTCGTCAGCGCCTCCACCGTCCAAACCGGCATCAACATCGCTGGCCGCATC<br>CTTGGCGTGTTGGGCGTCCCATTCGCAGGACAGCTTGCTAGTTTCTACTCCTTC
	GGCATCTACCTCGAGCCCATTCACGGCGTGCCAACCGTGAGGTTCAACTTCACCAACCCACAGAAC<br>ATCTCCGATCGCGGCACTGCGAACTACTCCCAACCATACGAGTCCCCAGGCCTC
	ACCAACGCTGCTTCCTGGGTCAGGTACAACCAGTTCCGCAGGGACCTCACCTTGGGTGTCTTGGAC<br>CTCGTGGCCCTCTTCCCAAGCTACGACACCCGCACCTACCCCATCAACACCTCT
	ACCGTGGACTTCGACTTCTTCGTGTCCCGTGGTGGCACCACCGTAAACAACTTCAGGTTCCTCAGG<br>ACCATGAACAGCGGTGACGAGCTCAAGTACGGCAACTTCGTGAGGCGTGCCTTC
	CAGGGTTTGGGTGACTCCTTCAGGGCCTATCAACAGTCTCTGGAGGACTGGCTCGAGAACCGCGAC<br>GACGCTAGGACCAGGAGCGTCCTCTACACCCAGTACATCGCTTTGGAATTGGAC
	CAGTTGAAGGACTCCGAGACTGAGTTGCCACCAGAGACTACTGAGCGTCCAAACTACGAGTCTTAC<br>AGCCACAGGTTGTCCCACATCGGCATCATCCTGCAGTCTAGGGTGAACGTGCCA
	GACATCATCCGCACCTCCATCCAAGGCCTCAGCGGCAACGGCGAGGTGTACATCGACAAGATCGA<br>GATCATCCCAGTGACCGCCACCTTCGAGGCCGAGTACGACTTGGAGAGGTAATGA
	GAATTCGGCCTCACTAGCCAGGAGATCCAGAGGTACTACGAGCGCCAAGTCGAGAGGACTAGGGA<br>CTACTCCGACTACTGCGTGGAGTGGTACAACACTGGTCTCAACAGCTTGAGGGGC
	CTCGTCGGCGAGTTGTGGCCCAGGGGCAGGGACCAATGGGAGATCTTCTTGGAACACGTCGAGCA<br>ACTCATCAACCAGCAGATCACCGAGAATGCCAGGAACACCGCTCTCGCTCGTCTC
	ACCACCCCATTCACCTTCACCCAGATCCAAGACATCATCCGCACCTCCATCCAAGGCCTCAGCGGC<br>AACGGCGAGGTGTACATCGACAAGATCGAGATCATCCCAGTGACCGCCACCTTC
cry1Aa	TTGGACATTGTGTCTCTCTTCCCGAACTATGACTCCAGAACCTACCCTATCCGTACAGTGTCCCAAC<br>TTACCAGAGAAATCTATACTAACCCAGTTCTTGAGAACTTCGACGGTAGCTTC
	GAGCAGTTGATCAACCAGAGGATCGAAGAGTTCGCCAGGAACCAGGCCATCTCTAGGTTGGAAGGA<br>TTGAGCAATCTCTACCAAATCTATGCAGAGAGCTTCAGAGAGTGGGAAGCCGAT
	AGCAACAGTTCCGTGAGCATCATCAGAGCTCCTATGTTCTCATGGATTCATCGTAGTGCTGAGTTCA<br>ACAATATCATTCCTTCCTCTCAAATCACCCAAATCCCATTGACCAAGTCTACT
	ACCCTGTCTTCCACCTTGTACAGAAGACCCTTCAATATCGGTATCAACAACCAGCAACTTTCCGTTCT<br>TGACGGAACAGAGTTCGCCTATGGAACCTCTTCTAACTTGCCATCCGCTGTT
	TACGTTCAAGCAGCTAATCTTCACCTCAGCGTGCTTCGAGACGTTAGCGTGTTTGGGCAAAGGTGG<br>GGATTCGATGCTGCAACCATCAATAGCCGTTACAACGACCTTACTAGGCTGATT
	ATGGACAACAACCCAAACATCAACGAATGCATTCCATACAACTGCTTGAGTAACCCAGAAGTTGAAG<br>TACTTGGTGGAGAACGCATTGAAACCGGTTACACTCCCATCGACATCTCCTTG
	CCTACTAACCCAGCTCTCCGCGAGGAAATGCGTATTCAATTCAACGACATGAACAGCGCCTTGACCA<br>CAGCTATCCCATTGTTCGCAGTCCAGAACTACCAAGTTCCTCTCTTGTCCGTG

	CAAAGATATCGTGTCAGGATTCGTTACGCATCTACCACTAACTTGCAATTCCACACCTCCATCGACG GAAGGCCTATCAATCAGGGTAACTTCTCCGCAACCATGTCAAGCGGCAGCAAC
	ATCATGGCCTCTCCAGTTGGATTCAGCGGGCCCGAGTTTACCTTTCCTCTCTATGGAACTATGGGAA ACGCCGCTCCACAACAACGTATCGTTGCTCAACTAGGTCAGGGTGTCTACAGA
	TTGCAATCCGGCAGCTTCAGAACCGTCGGTTTCACTACTCCTTTCAACTTCTCTAACGGATCAAGCG TTTTCACCCTTAGCGCTCATGTGTTCAATTCTGGCAATGAAGTGTACATTGAC
	TACAGAAAGAGCGGAACCGTTGATTCCTTGGACGAAATCCCACCACAGAACAACAATGTGCCACCC AGGCAAGGATTCTCCCACAGGTTGAGCCACGTGTCCATGTTCCGTTCCGGATTC
	CGTGGTTCTGCCCAAGGTATCGAAGGCTCCATCAGGAGCCCACACTTGATGGACACCTTGAACAGC ATAACTATCTACACCGATGCTCACAGAGGAGAGTATTACTGGTCTGGACACCAG
	GGAAACTACACCGACCACGCTGTTCGTTGGTACAACACTGGCTTGGAGCGTGTCTGGGGTCCTGAT TCTAGAGATTGGATTAGATACAACCAGTTCAGGAGAGAATTGACCCTCACAGTT
	TTCTCTAACGGATCAAGCGTTTTCACCCTTAGCGCTCATGTGTTCAATTCTGGCAATGAAGTGTACAT TGACCGTATTGAGTTTGTGCCTGCCGAAGTTACCTTCGAGGCTGAGTACTGA
	TCCTTGACACAGTTTCTGCTCAGCGAGTTCGTGCCAGGTGCTGGGTTCGTTCTCGGACTAGTTGACA TCATCTGGGGTATCTTTGGTCCATCTCAATGGGATGCATTCCTGGTGCAAATT
	AACCTTGGATCTGGAACTTCTGTCGTGAAAGGACCAGGCTTCACAGGAGGTGATATTCTTAGAAGAA CTTCTCCTGGCCAGATTAGCACCCTCAGAGTTAACATCACTGCACCACTTTCT
cry1Ab/c	TACGTTCAAGCAGCTAATCTTCACCTCAGCGTGCTTCGAGACGTTAGCGTGTTTGGGCAAAGGTGG GGATTCGATGCTGCAACCATCAATAGCCGTTACAACGACCTTACTAGGCTGATT
	TCCTTGGATAATCTCCAATCCAGCGATTTCGGTTACTTTGAAAGTGCCAATGCTTTTACATCTTCACT CGGTAACATCGTGGGTGTTAGAAACTTTAGTGGGACTGCAGGAGTGATTATC
	CCTACTAACCCAGCTCTCCGCGAGGAAATGCGTATTCAATTCAACGACATGAACAGCGCCTTGACCA CAGCTATCCCATTGTTCGCAGTCCAGAACTACCAAGTTCCTCTCTTGTCCGTG
	TTGGACATTGTGTCTCTCTTCCCGAACTATGACTCCAGAACCTACCCTATCCGTACAGTGTCCCAAC TTACCAGAGAAATCTATACTAACCCAGTTCTTGAGAACTTCGACGGTAGCTTC
	GGAAACTACACCGACCACGCTGTTCGTTGGTACAACACTGGCTTGGAGCGTGTCTGGGGTCCTGAT TCTAGAGATTGGATTAGATACAACCAGTTCAGGAGAGAATTGACCCTCACAGTT
	ACCTTGTCTTCCACCTTGTACAGAAGACCCTTCAATATCGGTATCAACAACCAGCAACTTTCCGTTCT TGACGGAACAGAGTTCGCCTATGGAACCTCTTCTAACTTGCCATCCGCTGTT
	AGCAACAGTTCCGTGAGCATCATCAGAGCTCCTATGTTCTCTTGGATACATCGTAGTGCTGAGTTCA ACAACATCATCGCATCCGATAGTATTACTCAAATCCCTGCAGTGAAGGGAAAC
	CGTGGTTCTGCCCAAGGTATCGAAGGCTCCATCAGGAGCCCACACTTGATGGACATCTTGAACAGC ATAACTATCTACACCGATGCTCACAGAGGAGAGTATTACTGGTCTGGACACCAG
	GAGCAGTTGATCAACCAGAGGATCGAAGAGTTCGCCAGGAACCAGGCCATCTCTAGGTTGGAAGGA TTGAGCAATCTCTACCAAATCTATGCAGAGAGCTTCAGAGAGTGGGAAGCCGAT
	TTTCTCTTCAACGGTTCTGTCATTTCAGGACCAGGATTCACTGGTGGAGACCTCGTTAGACTCAACA GCAGTGGAAATAACATTCAGAATAGAGGGTATATTGAAGTTCCAATTCACTTC
	CCATCCACATCTACCAGATATAGAGTTCGTGTGAGGTATGCTTCTGTGACCCCTATTCACCTCAACG TTAATTGGGGTAATTCATCCATCTTCTCCAATACAGTTCCAGCTACAGCTACC

	TCCTTGACACAGTTTCTGCTCAGCGAGTTCGTGCCAGGTGCTGGGTTCGTTCTCGGACTAGTTGACATCATCTGGGGTATCTTTGGTCCATCTCAATGGGATGCATTCCTGGTGCAAATT
	ATCATGGCCTCTCCAGTTGGATTCAGCGGGCCCGAGTTTACCTTTCCTCTCTATGGAACTATGGGAAACGCCGCTCCACAACAACGTATCGTTGCTCAACTAGGTCAGGGTGTCTACAGA
	AGATTCGAGTTCATTCCAGTTACTGCAACACTCGAGGCTGAGTACAACCTCGAAAGAGCCCAGAAGGCTGTAATGCCCTCTTCACCTCTACAAACCAGCTTGGACTCAAGACAAATGTGA
	TACAGAAAGAGCGGAACCGTTGATTCCTTGGACGAAATCCCACCACAGAACAACAATGTGCCACCCAGGCAAGGATTCTCCCACAGGTTGAGCCACGTGTCCATGTTCCGTTCCGGATTC
	ATGGACAACAACCCAAACATCAACGAATGCATTCCATACAACTGCTTGAGTAACCCAGAAGTTGAAGTACTTGGTGGAGAACGCATTGAAACCGGTTACACTCCCATCGACATCTCCTTG
	GACAGATTCGAGTTCATTCCAGTTACTGCAACACTCGAGGCTGAGTACAACCTCGAAAGAGCCCAGAAGGCTGTAATGCCCTCTTCACCTCTACAAACCAGCTTGGACTCAAGACAAATG
cry1Ac	CCTACTAACCCAGCTCTCCGCGAGGAAATGCGTATTCAATTCAACGACATGAACAGCGCCTTGACCACAGCTATCCCATTGTTCGCAGTCCAGAACTACCAAGTTCCTCTCTTGTCCGTG
	TACGTTCAAGCAGCTAATCTTCACCTCAGCGTGCTTCGAGACGTTAGCGTGTTTGGGCAAAGGTGGGGATTCGATGCTGCAACCATCAATAGCCGTTACAACGACCTTACTAGGCTGATT
	ATGGACAACAACCCAAACATCAACGAATGCATTCCATACAACTGCTTGAGTAACCCAGAAGTTGAAGTACTTGGTGGAGAACGCATTGAAACCGGTTACACTCCCATCGACATCTCCTTG
	ACCTTGTCTTCCACCTTGTACAGAAGACCCTTCAATATCGGTATCAACAACCAGCAACTTTCCGTTCTTGACGGAACAGAGTTCGCCTATGGAACCTCTTCTAACTTGCCATCCGCTGTT
	TTTCTCTTCAACGGTTCTGTCATTTCAGGACCAGGATTCACTGGTGGAGACCTCGTTAGACTCAACAGCAGTGGAAATAACATTCAGAATAGAGGGTATATTGAAGTTCCAATTCACTTC
	GAGCAGTTGATCAACCAGAGGATCGAAGAGTTCGCCAGGAACCAGGCCATCTCTAGGTTGGAAGGATTGAGCAATCTCTACCAAATCTATGCAGAGAGCTTCAGAGAGTGGGAAGCCGAT
	TCCTTGACACAGTTTCTGCTCAGCGAGTTCGTGCCAGGTGCTGGGTTCGTTCTCGGACTAGTTGACATCATCTGGGGTATCTTTGGTCCATCTCAATGGGATGCATTCCTGGTGCAAATT
	AGATTCGAGTTCATTCCAGTTACTGCAACACTCGAGGCTGAGTACAACCTTGAGAGAGCCCAGAAGGCTGTGAACGCCCTCTTTACCTCCACCAATCAGCTTGGCTTGAAAACTAACGTT
	GACAGATTCGAGTTCATTCCAGTTACTGCAACACTCGAGGCTGAGTACAACCTTGAGAGAGCCCAGAAGGCTGTGAACGCCCTCTTTACCTCCACCAATCAGCTTGGCTTGAAAACTAAC
	CCATCCACATCTACCAGATATAGAGTTCGTGTGAGGTATGCTTCTGTGACCCCTATTCACCTCAACGTTAATTGGGGTAATTCATCCATCTTCTCCAATACAGTTCCAGCTACAGCTACC
	GGAAACTACACCGACCACGCTGTTCGTTGGTACAACACTGGCTTGGAGCGTGTCTGGGGTCCTGATTCTAGAGATTGGATTAGATACAACCAGTTCAGGAGAGAATTGACCCTCACAGTT
	AGCAACAGTTCCGTGAGCATCATCAGAGCTCCTATGTTCTCTTGGATACATCGTAGTGCTGAGTTCAACAACATCATCGCATCCGATAGTATTACTCAAATCCCTGCAGTGAAGGGAAAC
	TACAGAAAGAGCGGAACCGTTGATTCCTTGGACGAAATCCCACCACAGAACAACAATGTGCCACCCAGGCAAGGATTCTCCCACAGGTTGAGCCACGTGTCCATGTTCCGTTCCGGATTC
	CGTGGTTCTGCCCAAGGTATCGAAGGCTCCATCAGGAGCCCACACTTGATGGACATCTTGAACAGCATAACTATCTACACCGATGCTCACAGAGGAGAGTATTACTGGTCTGGACACCAG

	TCCTTGGATAATCTCCAATCCAGCGATTTCGGTTACTTTGAAAGTGCCAATGCTTTTACATCTTCACT CGGTAACATCGTGGGTGTTAGAAACTTTAGTGGGACTGCAGGAGTGATTATC
	TTGGACATTGTGTCTCTCTTCCCGAACTATGACTCCAGAACCTACCCTATCCGTACAGTGTCCCAAC TTACCAGAGAAATCTATACTAACCCAGTTCTTGAGAACTTCGACGGTAGCTTC
	ATCATGGCCTCTCCAGTTGGATTCAGCGGGCCCGAGTTTACCTTTCCTCTCTATGGAACTATGGGAA ACGCCGCTCCACAACAACGTATCGTTGCTCAACTAGGTCAGGGTGTCTACAGA
Cry1A105_1	GGCTATGTTACCAAGGAGCTTGAGTACTTTCCTGAGACCGACAAAGTGTGGATCGAGATCGGTGAA ACCGAGGGAACCTTCATCGTGGACAGCGTGGAGCTTCTCTTGATGGAGGAATAA
	GTTTACAGAAAGAGCGGAACCGTTGATTCCTTGGACGAAATCCCACCACAGAACAACAATGTGCCAC CCAGGCAAGGATTCTCCCACAGGTTGAGCCACGTGTCCATGTTCCGTTCCGGA
	TTGTCCTTGACACAGTTTCTGCTCAGCGAGTTCGTGCCAGGTGCTGGGTTCGTTCTCGGACTAGTTG ACATCATCTGGGGTATCTTTGGTCCATCTCAATGGGATGCATTCCTGGTGCAA
	AATGAGGACCTCGGAGTCTGGGTCATCTTCAAGATCAAGACCCAAGACGGACACGCAAGACTTGGC AACCTTGAGTTTCTCGAAGAGAAACCATTGGTCGGTGAAGCTCTCGCTCGTGTG
	GTCACCCTCTCCGGAACTTTCGACGAGTGCTACCCTACCTACTTGTACCAGAAGATCGATGAGTCCA AACTCAAAGCCTTCACCAGGTATCAACTTAGAGGCTACATCGAAGACAGCCAA
	AGATGCGCTCCACACCTTGAGTGGAATCCTGACTTGGACTGCTCCTGCAGGGATGGCGAGAAGTGT GCCCACCATTCTCATCACTTCTCCTTGGACATCGATGTGGGATGTACTGACCTG
	GTGTACGTTCAAGCAGCTAATCTTCACCTCAGCGTGCTTCGAGACGTTAGCGTGTTTGGGCAAAGGT GGGGATTCGATGCTGCAACCATCAATAGCCGTTACAACGACCTTACTAGGCTG
	GACCTTGAAATCTACTCGATCAGGTACAATGCCAAGCACGAGACCGTGAATGTCCCAGGTACTGGTT CCCTCTGGCCACTTTCTGCCCAATCTCCCATTGGGAAGTGTGGAGAGCCTAAC
	GCCATGGACAACAACCCAAACATCAACGAATGCATTCCATACAACTGCTTGAGTAACCCAGAAGTTG AAGTACTTGGTGGAGAACGCATTGAAACCGGTTACACTCCCATCGACATCTCC
	GTTACTGACTATCACATTGACCAAGTGTCCAACTTGGTCACCTACCTTAGCGATGAGTTCTGCCTCG ACGAGAAGCGTGAACTCTCCGAGAAAGTTAAACACGCCAAGCGTCTCAGCGAC
	TTCAGCAACAGTTCCGTGAGCATCATCAGAGCTCCTATGTTCTCTTGGATACACCGTAGTGCTGAGT TCAACAACATCATTGCATCCGACAGCATTACTCAAATACCCTTGGTGAAAGCA
	CGTGAGAACCCTTGCGAGTTCAACAGAGGTTACAGGGACTACACACCACTTCCAGTTGGCTATGTTA CCAAGGAGCTTGAGTACTTTCCTGAGACCGACAAAGTGTGGATCGAGATCGGT
	CAGATCATGGCCTCTCCAGTTGGATTCAGCGGGCCCGAGTTTACCTTTCCTCTCTATGGAACTATGG GAAACGCCGCTCCACAACAACGTATCGTTGCTCAACTAGGTCAGGGTGTCTAC
	CCCCAAAGGTATCGTGCAAGAATCCGCTATGCCTCTACTACAAATCTCAGGATCTACGTGACTGTTG CAGGTGAAAGGATCTTTGCTGGTCAGTTCAACAAGACTATGGATACCGGTGAC
	ATTGGAAACTACACCGACCACGCTGTTCGTTGGTACAACACTGGCTTGGAGCGTGTCTGGGGTCCT GATTCTAGAGATTGGATTAGATACAACCAGTTCAGGAGAGAATTGACCCTCACA
	AGAACCTTGTCTTCCACCTTGTACAGAAGACCCTTCAATATCGGTATCAACAACCAGCAACTTTCCGT TCTTGACGGAACAGAGTTCGCCTATGGAACCTCTTCTAACTTGCCATCCGCT
	GAGAGGAATCTCTTGCAAGACTCCAACTTCAAAGACATCAACAGGCAGCCAGAACGTGGTTGGGGT GGAAGCACCGGGATCACCATCCAAGGAGGCGACGATGTGTTCAAGGAGAACTAC

	CATACACTTCAGTCAGGTACTACTGTTGTCAGAGGTCCAGGGTTTACAGGAGGAGACATTCTTCGTCGCACAAGTGGAGGACCCTTTGCTTACACTATTGTTAACATCAATGGCCAATTG
	GACAGGTTTGAATTGATTCCAGTTACTGCAACCCTCGAGGCTGAGTACAACCTTGAGAGAGCCCAGAAGGCTGTGAACGCCCTCTTTACCTCCACCAATCAGCTTGGCTTGAAAACTAAC
	GTTTTGGACATTGTGTCTCTCTTCCCGAACTATGACTCCAGAACCTACCCTATCCGTACAGTGTCCCAACTTACCAGAGAAATCTATACTAACCCAGTTCTTGAGAACTTCGACGGTAGC
	GAACAGAACAATCAGCGTTCCGTCCTGGTTGTGCCTGAGTGGGAAGCTGAAGTGTCCCAAGAGGTTAGAGTCTGTCCAGGTAGAGGCTACATTCTCCGTGTGACCGCTTACAAGGAGGGA
	GAACTTGAGGGACGTATCTTTACCGCATTCTCCTTGTACGATGCCAGAAACGTCATCAAGAACGGTGACTTCAACAATGGCCTCAGCTGCTGGAATGTGAAAGGTCATGTGGACGTGGAG
	TTGCAAGCCGACACCAACATCGCCATGATCCACGCCGCAGACAAACGTGTGCACAGCATTCGTGAGGCTTACTTGCCTGAGTTGTCCGTGATCCCTGGTGTGAACGCTGCCATCTTCGAG
	TACGGTGAGGGTTGCGTGACCATCCACGAGATCGAGAACAACACCGACGAGCTTAAGTTCTCCAACTGCGTCGAGGAAGAAATCTATCCCAACAACACCGTTACTTGCAACGACTACACT
	CCTTTGACATTCCAATCTTTTAGCTACGCAACTATCAACACAGCTTTTACATTCCCAATGAGCCAGAGTAGCTTCACAGTAGGTGCTGACACTTTCAGCTCAGGGAATGAAGTTTACATC
	GATCCTACTAACCCAGCTCTCCGCGAGGAAATGCGTATTCAATTCAACGACATGAACAGCGCCTTGACCACAGCTATCCCATTGTTCGCAGTCCAGAACTACCAAGTTCCTCTCTTGTCC
	ATTGAGCAGTTGATCAACCAGAGGATCGAAGAGTTCGCCAGGAACCAGGCCATCTCTAGGTTGGAAGGATTGAGCAATCTCTACCAAATCTATGCAGAGAGCTTCAGAGAGTGGGAAGCC
	AAGAGAGCAGAGAAGAAGTGGAGGGACAAACGTGAGAAACTCGAATGGGAAACTAACATCGTTTACAAGGAGGCCAAAGAGTCCGTGGATGCTTTGTTCGTGAACTCCCAATATGATCAG
	GTGAATCAGGAAGAGTACGGAGGTGCCTACACTAGCCGTAACAGAGGTTACAACGAAGCTCCTTCCGTTCCTGCTGACTATGCCTCCGTGTACGAGGAGAAATCCTACACAGATGGCAGA
	TTCCGTGGTTCTGCCCAAGGTATCGAAGGCTCCATCAGGAGCCCACACTTGATGGACATCTTGAACAGCATAACTATCTACACCGATGCTCACAGAGGAGAGTATTACTGGTCTGGACAC
Cry1A105_2	GTTCAAGGAGAACTACGTCACCCTCTCCGGAACTTTCGACGAGTGCTACCCTACCTACTTGTACCAGAAGATCGATGAGTCCAAACTCAAAGCCTTCACCAGGTATCAACTTAGAGGCTA
	GAATGAAGTTTACATCGACAGGTTTGAATTGATTCCAGTTACTGCAACCCTCGAGGCTGAGTACAACCTTGAGAGAGCCCAGAAGGCTGTGAACGCCCTCTTTACCTCCACCAATCAGCT
	CATCGAAGACAGCCAAGACCTTGAAATCTACTCGATCAGGTACAATGCCAAGCACGAGACCGTGAATGTCCCAGGTACTGGTTCCCTCTGGCCACTTTCTGCCCAATCTCCCATTGGGAA
	TTCGTGCCAGGCGCTGGCTTCGTCCTGGGCCTCGTGGACATCATCTGGGGCATCTTTGGCCCCTCCCAGTGGGACGCCTTCCTGGTGCAAATCGAGCAGCTCATCAACCAGAGGATCGAG
	AGCTCTCGCTCGTGTGAAGAGAGCAGAGAAGAAGTGGAGGGACAAACGTGAGAAACTCGAATGGGAAACTAACATCGTTTACAAGGAGGCCAAAGAGTCCGTGGATGCTTTGTTCGTGAA
	CAAGCGTCTCAGCGACGAGAGGAATCTCTTGCAAGACTCCAACTTCAAAGACATCAACAGGCAGCCAGAACGTGGTTGGGGTGGAAGCACCGGGATCACCATCCAAGGAGGCGACGATGT
	TGGCTTGAAAACTAACGTTACTGACTATCACATTGACCAAGTGTCCAACTTGGTCACCTACCTTAGCGATGAGTTCTGCCTCGACGAGAAGCGTGAACTCTCCGAGAAAGTTAAACACGC

CTACTGGTCCGGCCACCAGATCATGGCCTCCCCGGTCGGCTTCAGCGGCCCCGAGTTTACCTTTCCTCTCTACGGCACGATGGGCAACGCCGCTCCACAACAACGCATCGTCGCTCAGCT
ATGCGCATCCAGTTCAACGACATGAACAGCGCCCTGACCACCGCCATCCCACTCTTCGCCGTCCAGAACTACCAAGTCCCGCTCCTGTCCGTGTACGTCCAGGCCGCCAACCTGCACCTC
GAGTTCGCCAGGAACCAGGCCATCAGCCGCCTGGAGGGCCTCAGCAACCTCTACCAAATCTACGCTGAGAGCTTCCGCGAGTGGGAGGCCGACCCCACTAACCCAGCTCTCCGCGAGGAG
TTGCAACGACTACACTGTGAATCAGGAAGAGTACGGAGGTGCCTACACTAGCCGTAACAGAGGTTACAACGAAGCTCCTTCCGTTCCTGCTGACTATGCCTCCGTGTACGAGGAGAAATC
CTCCCAATATGATCAGTTGCAAGCCGACACCAACATCGCCATGATCCACGCCGCAGACAAACGTGTGCACAGCATTCGTGAGGCTTACTTGCCTGAGTTGTCCGTGATCCCTGGTGTGAA
TATGGATACCGGTGACCCTTTGACATTCCAATCTTTTAGCTACGCAACTATCAACACAGCTTTTACATTCCCAATGAGCCAGAGTAGCTTCACAGTAGGTGCTGACACTTTCAGCTCAGG
CTACACAGATGGCAGACGTGAGAACCCTTGCGAGTTCAACAGAGGTTACAGGGACTACACACCACTTCCAGTTGGCTATGTTACCAAGGAGCTTGAGTACTTTCCTGAGACCGACAAAGT
GGGATGTACTGACCTGAATGAGGACCTCGGAGTCTGGGTCATCTTCAAGATCAAGACCCAAGACGGACACGCAAGACTTGGCAACCTTGAGTTTCTCGAAGAGAAACCATTGGTCGGTGA
CATCAATGGCCAATTGCCCCAAAGGTATCGTGCAAGAATCCGCTATGCCTCTACTACAAATCTCAGGATCTACGTGACTGTTGCAGGTGAAAGGATCTTTGCTGGTCAGTTCAACAAGAC
CGCTTACAAGGAGGGATACGGTGAGGGTTGCGTGACCATCCACGAGATCGAGAACAACACCGACGAGCTTAAGTTCTCCAACTGCGTCGAGGAAGAAATCTATCCCAACAACACCGTTAC
TCATGTGGACGTGGAGGAACAGAACAATCAGCGTTCCGTCCTGGTTGTGCCTGAGTGGGAAGCTGAAGTGTCCCAAGAGGTTAGAGTCTGTCCAGGTAGAGGCTACATTCTCCGTGTGAC
GAACTTCGACGGTAGCTTCAGGGGCAGCGCCCAGGGCATCGAGGGCTCCATCAGGAGCCCACACCTGATGGACATCCTCAACAGCATCACTATCTACACCGATGCCCACCGCGGCGAGTA
GGCTATGTTACCAAGGAGCTTGAGTACTTTCCTGAGACCGACAAAGTGTGGATCGAGATCGGTGAAACCGAGGGAACCTTCATCGTGGACAGCGTGGAGCTTCTCTTGATGGAGGAATAA
CGCTGCCATCTTCGAGGAACTTGAGGGACGTATCTTTACCGCATTCTCCTTGTACGATGCCAGAAACGTCATCAAGAACGGTGACTTCAACAATGGCCTCAGCTGCTGGAATGTGAAAGG
CAACCTGCCCTCCGCTGTCTACCGCAAGAGCGGCACGGTGGATTCCCTGGACGAGATCCCACCACAGAACAACAATGTGCCCCCCAGGCAGGGTTTTTCCCACAGGCTCAGCCACGTGTC
GTGTGGAGAGCCTAACAGATGCGCTCCACACCTTGAGTGGAATCCTGACTTGGACTGCTCCTGCAGGGATGGCGAGAAGTGTGCCCACCATTCTCATCACTTCTCCTTGGACATCGATGT
AGCGTGCTGAGGGACGTCAGCGTGTTTGGCCAGAGGTGGGGCTTCGACGCCGCCACCATCAACAGCCGCTACAACGACCTCACCAGGCTGATCGGCAACTACACCGACCACGCTGTCCGC
GGGCCAGGGCGTCTACCGCACCCTGAGCTCCACCCTGTACCGCAGGCCCTTCAACATCGGTATCAACAACCAGCAGCTGTCCGTCCTGGATGGCACTGAGTTCGCCTACGGCACCTCCTC
ACCCTTGGTGAAAGCACATACACTTCAGTCAGGTACTACTGTTGTCAGAGGTCCAGGGTTTACAGGAGGAGACATTCTTCGTCGCACAAGTGGAGGACCCTTTGCTTACACTATTGTTAA
CATGTTCCGCTCCGGCTTCAGCAACTCGTCCGTGAGCATCATCAGAGCTCCTATGTTCTCTTGGATACACCGTAGTGCTGAGTTCAACAACATCATTGCATCCGACAGCATTACTCAAAT

	TGCATCCCGTACAACTGCCTCAGCAACCCTGAGGTCGAGGTGCTCGGCGGTGAGCGCATCGAGAC<br>CGGTTACACCCCCATCGACATCTCCCTCTCCCTCACGCAGTTCCTGCTCAGCGAG
	TGGTACAACACTGGCCGTCCTGGACATTGTGTCCCTCTTCCCGAACTACGACTCCCGCACCTACCC<br>GATCCGCACCGTGTCCCAACTGACCCGCGAAATCTACACCAACCCCGTCCTGGA
Cry2Ab2	AACTCCGTCCTGAACTCTGGTCGCACCACCATCTGCGACGCCTACAACGTCGCGGCGCATGATCCA<br>TTCAGCTTCCAGCACAAGAGCCTCGACACTGTTCAGAAGGAGTGGACGGAGTGG
	TCCATGATCCACCTGGCGCCCAATGATTACACCGGCTTCACCATCTCTCCAATCCACGCCACCCAAG<br>TGAACAACCAGACACGCACCTTCATCTCCGAGAAGTTCGGCAACCAGGGCGAC
	GCAAACGTGGAGGAGTTCAACCGCCAAGTGGACAACTTCCTCAACCCCAACCGCAATGCGGTGCCT<br>CTGTCCATCACTTCTTCCGTGAACACCATGCAACAACTGTTCCTCAACCGCTTG
	AAGAAGAACAACCACAGCCTGTACCTGGACCCCATCGTCGGCACGGTGGCCAGCTTCCTTCTCAAG<br>AAGGTCGGCTCTCTCGTCGGGAAGCGCATCCTCTCGGAACTCCGCAACCTGATC
	CTTGGCCTCCGGAGCGGCGCCTTCACGGCGCGTGGAAATTCTAACTACTTCCCCGACTACTTCATC<br>AGGAACATCTCTGGTGTTCCTCTCGTCGTCCGCAACGAGGACCTCCGCCGTCCA
	TCCCTGAGGTTCGAGCAGAACAACACCACCGCCAGGTACACCCTGCGCGGCAACGGCAACAGCTA<br>CAACCTGTACCTGCGCGTCAGCTCCATTGGCAACTCCACCATCAGGGTCACCATC
	TGCTCCACCTTCCTGCCGCCGCTGCTCACCCCGTTCGTGAGGTCCTGGCTCGACAGCGGCTCCGA<br>CCGCGAGGGCGTGGCCACCGTCACCAACTGGCAAACCGAGTCCTTCGAGACCACC
	CCTCAGTTCCAGATGCAAGGCTACCAGCTGCTCCTGCTGCCACTCTTTGCTCAGGCTGCCAACCTG<br>CACCTCTCCTTCATTCGTGACGTGATCCTCAACGCTGACGAGTGGGGCATCTCT
	AACATTGTTGGCCTCCCCGGCTCCACCACAACTCATGCTCTGCTTGCTGCCAGAGTGAACTACTCC<br>GGCGGCATCTCGAGCGGCGACATTGGTGCATCGCCGTTCAACCAGAACTTCAAC
	CTGGAGTTCAGGACCTACATGTTCCTGAACGTGTTCGAGTACGTCAGCATCTGGTCGCTCTTCAAGT<br>ACCAGAGCCTGCTGGTGTCCAGCGGCGCCAACCTCTACGCCAGCGGCTCTGGT
	CTGCACTACAACGAGATCAGGAACATCGCCTCTCCGTCCGGGACGCCCGGAGGTGCAAGGGCGTA<br>CATGGTGAGCGTCCATAACAGGAAGAACAACATCCACGCTGTGCATGAGAACGGC
	TGGTGGCCAGCAGCAACTCCGACGTCCCGCTGGACATCAACGTGACCCTGAACTCTGGCACCCAG<br>TTCGACCTCATGAACATCATGCTGGTGCCAACTAACATCTCGCCGCTGTACTGAT
	CCCCAACAAACTCAGAGCTTCACCAGCCAGGACTGGCCATTCCTGTATTCGTTGTTCCAAGTCAACT<br>CCAACTACGTCCTCAACGGCTTCTCTGGTGCTCGCCTCTCCAACACCTTCCCC
	GCAGCCACGCTGAGGACCTACCGCGACTACCTGAAGAACTACACCAGGGACTACTCCAACTATTGC<br>ATCAACACCTACCAGTCGGCCTTCAAGGGCCTCAATACGAGGCTTCACGACATG
	TTTCCATCTGGCTCCACCAACCTCATGCAAGACATCCTCAGGGAGACCGAGAAGTTTCTCAACCAGC<br>GCCTCAACACTGATACCCTTGCTCGCGTCAACGCTGAGCTGACGGGTCTGCAA
	AACGGGAGGGTGTACACAGCCACCAATGTGAACACGACGACCAACAATGATGGCGTCAACGACAAC<br>GGCGCCCGCTTCAGCGACATCAACATTGGCAACGTGGTGGCCAGCAGCAACTCC
Cry3A055	TACAACGTGGGTCTAGACAAGCTCCGCGGCAGCAGCTACGAGAGCTGGGTGAACTTCAACCGCTAC<br>CGCCGCGAGATGACCCTGACCGTGCTGGACCTGATCGCCCTGTTCCCCCTGTAC
	GTGGCAGGCCCCCGCTTCACCGGCGGCGACATCATCCAGTGCACCGAGAACGGCAGCGCCGCCA<br>CCATCTACGTGACCCCCGACGTGAGCTACAGCCAGAAGTACCGCGCCCGCATCCAC

	TACGCCAGCACCAGCCAGATCACCTTCACCCTGAGCCTGGACGGGGCCCCCTTCAACCAATACTACTTCGACAAGACCATCAACAAGGGCGACACCCTGACCTACAACAGCTTCAACCTG
	ATGACGGCCGACAACAACACCGAGGCCCTGGACAGCAGCACCACCAAGGACGTGATCCAGAAGGGCATCAGCGTGGTGGGCGACCTGCTGGGCGTGGTGGGCTTCCCCTTCGGCGGCGCC
	CTGGCCAGCTTCAGCACCCCTTTCGAGCTGAGCGGCAACAACCTCCAGATCGGCGTGACCGGCCTGAGCGCCGGCGACAAGGTGTACATCGACAAGATCGAGTTCATCCCCGTGAACTAG
	GACGTGCGCCTGTACCCCAAGGAGGTGAAGACCGAGCTGACCCGCGACGTGCTGACCGACCCCATCGTGGGCGTGAACAACCTGCGCGGCTACGGCACCACCTTCAGCAACATCGAGAAC
	TACATCCGCAAGCCCCACCTGTTCGACTACCTGCACCGCATCCAGTTCCACACGCGTTTCCAGCCCGGCTACTACGGCAACGACAGCTTCAACTACTGGAGCGGCAACTACGTGAGCACC
	AACCTGGCCGTGTGGCCCTCTGCAGTGTACAGCGGCGTGACCAAGGTGGAGTTCAGCCAGTACAACGACCAGACCGACGAGGCCAGCACCCAGACCTACGACAGCAAGCGCAACGTGGGC
	AAGGCACTGGCCGAGCTACAGGGCCTCCAGAACAACGTGGAGGACTATGTGAGCGCCCTGAGCAGCTGGCAGAAGAACCCCGCTGCACCGTTCCGCAACCCCCACAGCCAGGGCCGCATC
	ACCATCCCCGTGCTGACCTGGACCCACAAGAGCGTCGACTTCTTCAACATGATCGACAGCAAGAAGATCACCCAGCTGCCCCTGGTGAAGGCCTACAAGCTCCAGAGCGGCGCCAGCGTG
	CTGGTGAGCTTCTACACCAACTTCCTGAACACCATCTGGCCCAGCGAGGACCCCTGGAAGGCCTTCATGGAGCAGGTGGAGGCCCTGATGGACCAGAAGATCGCCGACTACGCCAAGAAC
	CTGCTGAAGGACGCCCAAATCTACGGAGAGGAGTGGGGCTACGAGAAGGAGGACATCGCCGAGTTCTACAAGCGCCAGCTGAAGCTGACCCAGGAGTACACCGACCACTGCGTGAAGTGG
	CGCCCCAGCATCGGCAGCAACGACATCATCACCAGCCCCTTCTACGGCAACAAGAGCAGCGAGCCCGTGCAGAACCTTGAGTTCAACGGCGAGAAGGTGTACCGCGCCGTGGCTAACACC
	GCCGTGAGCTGGGACAGCATCGACCAGCTGCCCCCCGAGACCACCGACGAGCCCCTGGAGAAGGGCTACAGCCACCAGCTGAACTACGTGATGTGCTTCCTGATGCAGGGCAGCCGCGGC
	CGCGAGCTGTTCAGCCAGGCCGAGAGCCACTTCCGCAACAGCATGCCCAGCTTCGCCATCAGCGGCTACGAGGTGCTGTTCCTGACCACCTACGCCCAGGCCGCCAACACCCACCTGTTC
Cry34Ab1	CAGCCTCTACTTCGACAACCCGTTCGCCGGCTCCAACAAATACGACGGCCACTCCAACAAGTCCCAGTACGAGATCATCACCCAGGGCGGCTCCGGCAACCAGTCCCACGTGACCTACAC
	CAGTACGAGATCATCACCCAGGGCGGCTCCGGCAACCAGTCCCACGTGACCTACACCATCCAGACCACCTCCTCCCGCTACGGCCACAAGTCCTGAGTCATGAGTCATGAGTCAGTTAAC
	GTCGACTCTAGAGGATCCACACGACACCATGTCCGCCCGCGAGGTGCACATCGACGTGAACAACAAGACCGGCCACACCCTCCAGCTGGAGGACAAGACCAAGCTCGACGGCGGCAGGTG
	GCGCACCTCCCCGACCAACGTGGCCAACGACCAGATCAAGACCTTCGTGGCCGAATCCAACGGCTTCATGACCGGCACCGAGGGCACCATCTACTACTCAATTAATGGCGAGGCCGAGAT
Cry35Ab1	TGGGGCACCGAGATCGACCAGAAGACCACCATCATCAACACCCTCGGCTTCCAGATCAACATCGACAGCGGCATGAAGTTCGACATCCCGGAGGTGGGCGGCGGTACCGACGAGATCAAG
	AAGAACACCCAGATCAAGACCACCCCGTACTACATCCTCAAGAAGTACCAGTACTGGCAGAGGGCCGTGGGCTCCAACGTCGCGCTCCGCCCGCACGAGAAGAAGTCCTACACCTACGAG
	AACAACGACAAGATTAATGTGTCAACCTACTCCTCCACCAACTCCATCCAGAAGTGGCAGATCAAGGCCAACGGCTCCTCCTACGTGATCCAGTCCGACAACGGCAAGGTGCTCACCGCC

	GGCACCGGCCAGGCCCTCGGCCTCATCCGCCTCACCGACGAGTCCTCCAACAACCCGAACCAGCA ATGGAACCTGACGTCCGTGCAGACCATCCAGCTCCCGCAGAAGCCGATCATCGAC
	CTCACCATCACCTCCCTGGAGCTCTACCGCTACAACGGCTCCGAGATCCGCATCATGCAGATCCAG ACCTCCGACAACGACACCTACAACGTGACCTCCTACCCGAACCACCAGCAGGCC
	ATGAACAAGAACGACGACGACATCGACGACTACAACCTCAAGTGGTTCCTCTTCCCGATCGACGAC GACCAGTACATCATCACCTCCTACGCCGCCAACAACTGCAAGGTGTGGAACGTG
	CTCTACCGCTACAACGGCTCCGAGATCCGCATCATGCAGATCCAGACCTCCGACAACGACACCTAC AACGTGACCTCCTACCCGAACCACCAGCAGGCCCTGCTGCTGACCAACCAC
	ACCCAGCTCAACGAGGAGCTCAAGATCGAGTATTCACATGAGACGAAGATCATGGAGAAGTACCAG GAGCAGTCCGAGATCGACAACCCGACCGACCAGTCCATGAACTCCATCGGCTTC
	ACCAAGCTCAAGGACTACCCGAAGTACTCCCCGACCGGCAACATCGACAACGGCACCTCCCCGCA GCTCATGGGCTGGACCCTCGTGCCGTGCATCATGGTGAACGACCCGAACATCGAC
Epsps_1	GAAGGCGGCGGCGACAGCGAGAATCGGATATTCGTCGATCATCGAAGGCGCGCGGTCTTCCGGCA CCGTGACGCCCTTCAGCGTGGAGGAGCGAACGCGCAGGTCCGCCACGTCTTCGCC
	AAGCAGGGCCGCAACCAGCGGGAAGGCCGTCGAGGACGGGTCGCCCGGCACGTCGATGACTTGG CCGGTGAGCTTGCCGCGGCCTTCCAGGCGGATGGTGCGCACGCCGTCCGCATCCGT
	GCCGGCAAGGCGCGGGTTGATGACTTCGATGTCGGCGCCCATTTCCTGCAGCGTCAGGATGAGGC CGGTGCGGGTGGGGTTCATCAGCACGTTGAGGATGGTGACGTCGGAGCCCGGAAC
	CTCGACGGTAAGGTTGGCGCCAAAGCCCTGCAGCATCTTTTCCGTATGATCGCGCGTCATGATCGG CTCGATGACCGTCGTGATGCCGGGCGTGTTGAGGCCGGCGAGCAGCACGGCGGA
	CTTCACCTGTGCGGAGGCCATCGGCACGCGGTAGGTGATCGGCGTCGGCGTCTTCGGCCCGCGCA AGGTAACGGGAAGACGGTCACCGTCTTCCGATTTCACCTGCACGCCCATTTCGCG
	GCCGAAATCGAGCGGCGCCTCAGGCGCCAGGAGGCCGCCATTGCCGACGCCATCGATGATCCAG GTGTCGCCTTCCTTACGGATCCTGGCGCCCATGGCCTGCATGGCCTTGCCCGTATT
	GATGACGTCCTCGCCTTCCAGAAGGCCGGTGATGCGCGTTTCACCGCTCGCGAGACCGCCGAACA TGAAGGACCGGTGGGAGATCGACTTGTCGCCGGGAATGCGGACGGTTCCGGAAAG
	CGACACGAGGCCCATGACGAGGAAGCTCATGGCGATGCGGTGATCGAGATGGGTGGCGACGGCG GCGCCCGAGGCGTTGCCGAGCCCCTTGCCGTCAGGGCGGCCACGCACGACGAGCGA
	TCATCAGGCAGCCTTCGTATCGGAGAGTTCGATCTTCGCGCCCAGCCCGGCCATCAGGTCCATGAA CTCCGGGAAGCTCGTGGCGATCATCGTGGCATCGTCCACCGTGACAGGGTTTTC
	CGGGAATGCGGACGGTTCCGGAAAGGCCAGAGGATTTGCGGGCGGTTGCGGGCCGGCTGCTTGC ACCGTGAAGCATGCAGGCTGTAGCCACTGATGCTGAAATCCTAAAGGAACAAAACT
	CGTCTCGCCCTCATCGCAATCCACGCCATTGAGCTTGAGGCCATTGGCGACGGCCGAGAGGCGGT CGCTTTCCTTGACGCGGAGTTCTTCCAGACCGTTCATCACGGTCGCCCCTTCCGC
	CAGCGGGTTCAACACGCGGCCCATCGGGCGCTTTGTGAGCGAGGCGTCGCCGATGAAGGTGCTGT CGAAATCGTAGACCCCGACGAGGCCCATGGTCAGGCGGCAGCCCGTGGCGGCATT
EPSPS_2	TCTTCACCTTCCAAAAGACCGGTGATACGGGTTTCACCGCTAGCGAGACCTCCAAACATGAAGGAC CTGTGGGAGATAGACTTGTCACCTGGAATACGGACGGTTCCAGAAAGACCAGAG
	CCTTCATCGCAATCAACACCGTTGAGCTTGAGACCGTTTGCGACAGCAGAAAGACGGTCGCTTTCCT TAACACGGAGTTCTTCCAAACCGTTCATAACGGTAGCACCTTCAGCGAATGCA

	GCAGCAACCAATGGGAAAGCAGTAGAGGATGGATCACCTGGAACATCAATCACTTGACCGGTGAGC<br>TTACCACGACCTTCAAGACGGATGGTACGCACACCGTCAGCATCAGTCTCAACG
	TTCAACACACGACCCATTGGACGCTTAGTGAGAGAAGCGTCACCAATGAAAGTGCTATCGAAATCGT<br>AAACACCAACAAGACCCATAGTCAAACGGCAACCAGTTGCAGCGTTACCGAAA
	ACCTCCAAACATGAAGGACCTGTGGGAGATAGACTTGTCACCTGGAATACGGACGGTTCCAGAAAG<br>ACCAGAGGACTTACGAGCAGTTGCTGGACGGCTGCTTGCACCGTGAAGCATGCA
	TGAGCGGAAGCCATAGGTACCCTGTAGGTGATTGGCGTTGGAGTCTTTGGTCCACGCAAGGTAACT<br>GGAAGACGATCACCGTCTTCAGACTTCACCTGCACACCCATTTCGCGAAGTGGG
	GCTGCAACAGCGAGAATTGGATACTCGTCGATCATAGAAGGAGCACGGTCTTCTGGAACAGTAACA<br>CCCTTCAAAGTAGAAGAACGAACACGCAAGTCAGCCACGTCTTCTCCACCAGCA
	GTAAGGTTAGCACCAAAACCTTGAAGCATCTTTTCAGTGTGGTCACGAGTCATGATTGGCTCGATAA<br>CAGTGGTGATACCTGGGGTGTTGAGACCAGCAAGCAGAACAGCGGACTTCACT
	TCGAGAGGAGCCTCAGGAGCAAGGAGTCCACCGTTACCAACACCATCAATGATCCAAGTATCACCT<br>TCCTTACGGATTCTGGCACCCATAGCTTGCATAGCCTTACCAGTGTTGATAACA
	GCAGCCTTAGTGTCGGAGAGTTCGATCTTAGCTCCAAGACCAGCCATCAAATCCATGAACTCTGGG<br>AAGCTAGTAGCGATCATAGTAGCATCATCAACAGTAACAGGGTTTTCAGAAACG
	AGACCCATAACGAGGAAGCTCATAGCGATACGGTGATCGAGGTGGGTAGCGACAGCTGCTCCAGA<br>AGCGTTACCGAGACCCTTACCGTCAGGACGACCACGCACGACGAGAGAAGTCTCA
	AGACGTGGGTTGATCACTTCGATGTCGGCACCCATTTCCTGCAGAGTCAAGATGAGACCAGTACGG<br>GTTGGGTTCATCAAAACGTTAAGGATGGTGACGTCGGAACCTGGAACAAGCAAG
2mEPSPS	CGACGACCACAGGATGGCGATGGCTTTCTCCCTTGCCGCCTGTGCCGAGGTCCCCGTCACCATCC<br>GGGACCCTGGGTGCACCCGGAAGACCTTCCCCGACTACTTCGATGTGCTGAGCAC
	GGCCGGCGCCGAGGAGATCGTGCTGCAGCCCATCAAGGAGATCTCCGGCACCGTCAAGCTGCCG<br>GGGTCCAAGTCGCTTTCCAACCGGATCCTCCTACTCGCCGCCCTGTCCGAGGGGAC
	GCAGGGTGATGTGAAGTTTGCTGAGGTACTGGAGATGATGGGAGCGAAGGTTACATGGACCGAGA<br>CTAGCGTAACTGTTACTGGCCCACCGCGGGAGCCATTTGGGAGGAAACACCTCAA
	CGTGCTTGATGGAGTACCAAGAATGAGGGAGAGACCCATTGGCGACTTGGTTGTCGGATTGAAGCA<br>GCTTGGTGCAGATGTTGATTGTTTCCTTGGCACTGACTGCCCACCTGTTCGTGT
	ATACAAGTCCCCTAAAAATGCCTATGTTGAAGGTGATGCCTCAAGCGCAAGCTATTTCTTGGCTGGT<br>GCTGCAATTACTGGAGGGACTGTGACTGTGGAAGGTTGTGGCACCACCAGTTT
	GATGGCTTTCTCCCTTGCCGCCTGTGCCGAGGTCCCCGTCACCATCCGGGACCCTGGGTGCACCC<br>GGAAGACCTTCCCCGACTACTTCGATGTGCTGAGCACTTTCGTCAAGAATTAAGC
	TGGTGGAAAGTTCCCAGTTGAGGATGCTAAAGAGGAAGTGCAGCTCTTCTTGGGGAATGCTGGAAT<br>CGCAATGCGGTCCTTGACAGCAGCTGTTACTGCTGCTGGTGGAAATGCAACTTA
	CAATGGAATCGGAGGGCTACCTGGTGGCAAGGTCAAGCTGTCTGGCTCCATCAGCAGTCAGTACTT<br>GAGTGCCTTGCTGATGGCTGCTCCTTTGGCTCTTGGGGATGTGGAGATTGAAAT
	CATTGATAAATTAATCTCCATTCCGTACGTCGAAATGACATTGAGATTGATGGAGCGTTTTGGTGTGA<br>AAGCAGAGCATTCTGATAGCTGGGACAGATTCTACATTAAGGGAGGTCAAAA
	AACAGTGGTTGATAACCTGCTGAACAGTGAGGATGTCCACTACATGCTCGGGGCCTTGAGGACTCT<br>TGGTCTCTCTGTCGAAGCGGACAAAGCTGCCAAAAGAGCTGTAGTTGTTGGCTG

	CGAGAGGATGGTTGCGATCCGGACGGAGCTAACCAAGCTGGGAGCATCTGTTGAGGAAGGGCCGG ACTACTGCATCATCACGCCGCCGGAGAAGCTGAACGTGACGGCGATCGACACGTA
	GGCGATTGATGTCAACATGAACAAGATGCCTGATGTCGCCATGACTCTTGCTGTGGTTGCCCTCTTT GCCGATGGCCCGACAGCCATCAGAGACGTGGCTTCCTGGAGAGTAAAGGAGAC
bar	CTGCGGGCGGCCGGCTTCAAGCACGGGAACTGGCATGACGTGGGTTTCTGGCAGCTGGACTTCAG CCTGCCGGTACCGCCCCGTCCGGTCCTGCCCGTCACCGAGATCTGAACGGAGTGC
	TACATCGAGACAAGCACGGTCAACTTCCGTACCGAGCCGCAGGAACCGCAGGAGTGGACGGACGA CCTCGTCCGTCTGCGGGAGCGCTATCCCTGGCTCGTCGCCGAGGTGGACGGCGAG
	AGTGCGCGTGGGCATCGCCCGAGTTGGAGCTGGTACGGGAACTCATCGAACTCAACTGGCATACC CGCAATGGTGAGGTGGAACCGCGGCGGATCGCGTACGACCGTGCCCAGGAGGCCT
	ACCCACCTGCTGAAGTCCCTGGAGGCACAGGGCTTCAAGAGCGTGGTCGCTGTCATCGGGCTGCC CAACGACCCGAGCGTGCGCATGCACGAGGCGCTCGGATATGCCCCCCGCGGCATG
	GCTCGCTGTCATTTTCGAGACGCCATCTTTGGAAGCGGTGGCCGAATCCGTACTGCGCGGACTCGA CGACGCGTAAAACGATCGACCACGTACACGAGTCCGGACACGGGGCGAGGAGGC
	GTCGCCGGCATCGCCTACGCGGGCCCCTGGAAGGCACGCAACGCCTACGACTGGACGGCCGAGT CGACCGTGTACGTCTCCCCCCGCCACCAGCGGACGGGACTGGGCTCCACGCTCTAC
	CCGGTTCCGGCACCGAGGAAGACCGAAGGAAGACCACACGTGAGCCCAGAACGACGCCCGGCCG ACATCCGCCGTGCCACCGAGGCGGACATGCCGGCGGTCTGCACCATCGTCAACCAC
Pat	CATGTCTCCGGAGAGGAGACCAGTTGAGATTAGGCCAGCTACAGCAGCTGATATGGCCGCGGTTTG TGATATCGTTAACCATTACATTGAGACGTCTACAGTGAACTTTAGGACAGAGCC
	GTCTGTGGTTGCTGTTATAGGCCTTCCAAACGATCCATCTGTTAGGTTGCATGAGGCTTTGGGATAC ACAGCCCGGGGTACATTGCGCGCAGCTGGATACAAGCATGGTGGATGGCATGA
	GAACGCTTACGATTGGACAGTTGAGAGTACTGTTTACGTGTCACATAGGCATCAAAGGTTGGGCCTA GGATCCACATTGTACACACATTTGCTTAAGTCTATGGAGGCGCAAGGTTTTAA
	ACAAACACCACAAGAGTGGATTGATGATCTAGAGAGGTTGCAAGATAGATACCCTTGGTTGGTTGCT GAGGTTGAGGGTGTTGTGGCTGGTATTGCTTACGCTGGGCCCTGGAAGGCTAG
	CAGCTGGATACAAGCATGGTGGATGGCATGATGTTGGTTTTTGGCAAAGGGATTTTGAGTTGCCAG CTCCTCCAAGGCCAGTTAGGCCAGTTACCCAGATCTGAGTCGACCTGCAGGCAT
Prsv_cp_1	GCCAAGACTTTCTTTAGTCCGCTGATGGGACACTATATGAAAAGCGTTCTAAGCAAGGAAGCATACG TTAAGGACTTATTGAAATACTCAAGTGACATAATTGTTGGTGAAGTTGACCAC
	TCATTCTCTGGTTCATTTGCTGAGCTTGGACTTAAGTACGATTTCACTCAAAGGCACCGAAATAAACA AGATTTGTGGTTCATGTCACATCGAGGTATTCTGATCGATGACATCTATATC
	ACGCACCAGATCCGCAGGTTCTATCAATGGGTGCTTGAGCAGGCTCCATTCAATGAATTAGCGAAG CAGGGCAGGGCTCCATATGTGTCTGAGGTTGGATTGAGGCGCTTATACACTAGT
	ATATTTTGAGCGAGAAAGGGGAGATTCGCCTGAGCTACTGGTGTATTATGAATCGAGAAGCACTGAT GATCATCAGTTAACTTGCGGCAGTAAAACACATGTGTTTCACCAGTCTAAGTA
	AATGGATCATTAAAGGCCGAGCTGCGACCCGCAGAAAAAGTTCTAGCCAAAAAGACAAGATCTTTCA CTGCGGCGCCTCTTGACACTTTGCTGGGAGCCAAAGTTTGTGTTGATGATTTC
	GTTGGGGCTTTGTACAAAGGAAAGAAAAGAAAATATTTCGAAGGATCAACGGTTGAGCACAGACATA CTTTGGTGCGGAAAAGTTGTGAGCGTTTGTATGAAGGGCGAATGGGTGTGTGG

	AATAATTGGTTCTACAGCAAAAATATGGAGTGTCCTTGGACTGTCGGCATGACAAAGTTTTATAAAGGCTGGGACGAATTCTTGAGAAAATTTCCTGATGGCTGGGTTTATTGTGATGCA
	GAAATTACATACACACCTATTTTGACACCTGACGGGACGATTGTCAAGAAATTCAAAGGGAATAATAGTGGTCAACCTTCAACAGTTGTCGATAACACACTGATGGTTTTAATCACAATG
	TATTACGCGCTACGCAAGGCTGGTTACGACACGAAGGCTCAGGAGGACATGTGTGTGTTTTATATCAATGGTGATGATCTCTGTATTGCCATCCACCCGGATCATGAACATGTTCTTGAC
	GATGGATCTCAGTTTGATAGCTCTTTGACACCATATTTGCTTAATGCTGTGTTATCAATTCGACTGTGGGCTATGGAAGATTGGGATATTGGAGCTCAGATGCTTAAGAATCTGTATGGA
	GATGTTTTTGAGGAAAGTGTTGCACAAGTTGTCGAATTGCTAAACGACCACGAGTGTCCTGAACTCGAATACATCACAGATAGCGAGGTTATCATACAAGCTCTAAATATGGATGCTGCT
	ATGGATAAGTTACACGGCAATCTTAAGGGAGTGAGCTCAGCTTCTAGCAATTTAGTCACGAAGCATGTTGTGAAAGGCATTTGTCCCCTCTTCCGTAGTTACCTGGAGTGTAATGAGGAA
	AAGCGTGGGTCAATGGATGAATTGGAGGCCTACATAGATAAATATTTTGAGCGAGAAAGGGGAGATTCGCCTGAGCTACTGGTGTATTATGAATCGAGAAGCACTGATGATCATCAGTTA
	CCCAAACTTGAACCTGAGAGAATTGTGGCCATCCTTGAGTGGGATAAATCCAAGCTTCCAGAACATCGATTGGAAGCTATCACAGCAGCAATGATAGAGTCATGGGGATATGAGGAGCTA
Prsv_cp_2	GGCGTCTAAAAATGAAGCTGTGGATACCGGTCTGAATGAGAAGCTCAAAGAAAAAGAAAAGCAGAAAGAAAAAGAAAAAGATAAACAACAAGATAAAGACAATGATGGAGCTAGTGACGG
	GCACTCTCTCCTGGGTATGCGCAATTGAATACTCGCGCTAGTGTGTTTGTCGGGCCTGGCTCGACCCTGTTTCACCTTATAATACTATGTAAGCATTAGAATATAGTGTGGCTGCGCCAC
	AAACGATGTGTCAACTAGCACAAAAACTGGAGAGAGAGATAGGGATGTCAATGCCGGAACTAGTGGAACCTTCACTGTTCCGAGGATAAAGTCATTTACTGATAAGATGATCTTACCAAG
	GAAGTACATGCCGCGGTATGGAATCAAGAGAAATTTGACTGACATTAGTCTCGCTAGATATGCTTTCGATTTCTATGAGGTGAATTCGAAAACACCTGATAGGGCTCGTGAAGCTCATAT
	AAATGATTATGGCCTTAATGATAACGAAATGCAAGTAATGTTAAATGGTTTGATGGTTTGGTGTATCGAAAATGGTACATCTCCAGATATATCTGGTGTCTGGGTTATGATGGATGGGGA
	GCTGCGCCACCGCTTCTATTTTACAGTGAGGGTAGCCCTCCGTGCTTTTAGTGTTATTCGAGTTCTCTGAGTCTCCATACAGTGTGGGTGGCCCACGTGCTATTCGAGCCTCTTGGAATG
	GCAGATGAAGGCTGCAGCGCTACGCAATACTAATCGCAAAATGTTTGGAATGGACGGCAGTGTCAGTAACAAGGAAGAAAACACGGAGAGACACACAGTGGAAGATGTCAACAGAGACAT
	AATTAAGGGAAAAACTGTCCTTAATTTAAATCATCTTCTTCAGTATAATCCGAAACAAGTTGACATCTCAAACACTCGCGCCACTCAATCTCAATTTGAGAAGTGGTATGAGGGAGTGAG
	AACCCAAGTCGATTATCCCATTAAACCTTTGATTGAACACGCAACTCCTTCATTTAGGCAAATCATGGCTCACTTCAGTAACGCGGCAGAGGCATACATCGCGAAGAGGAATGCAACTGA
Hsp70	GAAATTTTGATGTTTATCTCTGCTCCTTTATTGTGACCATAAGTCAAGATCAGATGCACTTGTTTTAAATATTGTTGTCTGAAGAAATAAGTACTGACAGTATTTTGATGCATTGATCTG
	ACTGACACTATATTGCTTCTCTTTACATACGTATCTTGCTCGATGCCTTCTCCCTAGTGTTGACCAGTGTTACTCACATAGTCTTTGCTCATTTCATTGTAATGCAGATACCAAGCGGCC
	GCGGTATTTATTTAAGCACATGTTGGTGTTATAGGGCACTTGGATTCAGAAGTTTGCTGTTAATTTAGGCACAGGCTTCATACTACATGGGTCAATAGTATAGGGATTCATATTATAGGC

CGTCTTCGGTACGCGCTCACTCCGCCCTCTGCCTTTGTTACTGCCACGTTTCTCTGAATGCTCTCTTGTGTGGTGATTGCTGAGAGTGGTTTAGCTGGATCTAGAATTACACTCTGAAAT
GATACTATAATAATTTGTTCGTCTGCAGAGCTTATTATTTGCCAAAATTAGATATTCCTATTCTGTTTTTGTTTGTGTGCTGTTAAATTGTTAACGCCTGAAGGAATAAATATAAATGAC
CTTGTTTGTTGTAACAAAATTTAAAAATAAAGAGTTTCCTTTTTGTTGCTCTCCTTACCTCCTGATGGTATCTAGTATCTACCAACTGACACTATATTGCTTCTCTTTACATACGTATCT
CGTGTTCTGCCTGTGCTGATTACTTGCCGTCCTTTGTAGCAGCAAAATATAGGGACATGGTAGTACGAAACGAAGATAGAACCTACACAGCAATACGAGAAATGTGTAATTTGGTGCTTA

Detection and identification of transgenic events by next generation sequencing combined with enrichment technologies

Frédéric Debode, Julie Hulin, Benoît Charloteaux, Wouter Coppieters, Marc Hanikenne, Latifa Karim and Gilbert Berben

Supplementary Data S2. Commands and parameters used for the bioinformatic workflow

```
# RMQ : REF_GENOME = database used for enrichment, preformated with the command "bwa index REF_GENOME"

#### First part of the analysis : Analysis by counts
# Perform alignments
bwa mem -M -t 8 REF_GENOME File_sample01_R1.fastq.gz File_sample01_R2.fastq.gz | \
samtools view -bS - | samtools sort -o RESULT_BWA_sample01.sorted.bam
# Index the sequences
samtools index RESULT_BWA_sample01.sorted.bam
# Make count file
samtools idxstats RESULT_BWA_sample01.sorted.bam > RESULT_BWA_sample01.sorted.bam.counts
# The count file is processed with R, to convert raw counts in RPKM values and calculate the LOD

#### Second part of the analysis : make contigs
# Extract only sequences aligned against sequences present in the database
# (all the header of these sequences must be provided in the file "LIST_SEQ_DB")
samtools view RESULT_BWA_sample01.sorted.bam LIST_SEQ_DB | cut -f1  | sort | uniq >SAMPLE_01.id
seqtk subseq File_sample01_R1.fastq.gz SAMPLE_01.id >SAMPLE_01.R1.unic.fastq
seqtk subseq File_sample01_R2.fastq.gz SAMPLE_01.id >SAMPLE_01.R2.unic.fastq

# In fastq files, sequences identifiers are modified with a perl oneliner to distinguish reads R1 and R2
perl -i.bak -nle '~ s/(^@[M].+)/$1\/1/ ; print' SAMPLE_01.R1.unic.fastq
perl -i.bak -nle '~ s/(^@[M].+)/$1\/2/ ; print' SAMPLE_01.R2.unic.fastq

# Contigs are assembled with two programs in parallel
# First, assembly with SPADES
    spades.py --pe1-1 SAMPLE_01.R1.unic.fastq --pe1-2 SAMPLE_01.R2.unic.fastq -o spades/

# Second, assembly with MIRA
    # The program needs to write parameters in a "manifest" file.
            ########## EXAMPLE OF MANIFEST FILE ########################
            project = Enrichment
            job = est,denovo,accurate
            readgroup = SAMPLE_NAME
            data = SAMPLE_01.R1.unic.fastq SAMPLE_01.R2.unic.fastq
            technology = solexa
            template_size = 0 350 autorefine
            segment_placement = ---> <---
            segment_naming = solexa
            parameters =    -GENERAL :number_of_threads= 8 \
                            SOLEXA_SETTINGS \
            ########## END OF EXAMPLE MANIFEST FILE ####################
    # run mira
    mira manifest

# Third, integrate all contigs obtained with CISA
    # Firstly use merge.py ; to merge all contigs
        # Need to create "merge.config" file, with parameters
            ########## EXAMPLE OF MERGE.CONFIG FILE ####################
            count=2
            data=mira_contigs.fa,title=mira
            data=spades_contigs.fa,title=spades
            min_length=100
            Master_file=SAMPLE01_CISA.fa
            ########## END EXAMPLE OF MERGE.CONFIG FILE ################

        # Run first step of cisa
        python Merge.py merge.config

        # The terminal shows important information for the following step.
        # Note the largest "Whole" value. For our example, we use 123456 as being this largest "Whole" value
    # Secondly for CISA
        # Need to create "cisa.config" file
            ########## EXAMPLE OF CISA.CONFIG FILE #####################
            genome=123456
            infile=SAMPLE01_CISA.fa
            outfile=SAMPLE01_CONTIG_CISA.fa
            R2_Gap=0.95
            CISA=<PATH_TO_CISA_DIRECTORY>
            makeblastdb=/usr/bin/makeblastdb
            blastn=/usr/bin/blastn
            nucmer=/usr/bin/nucmer
            ########## END EXAMPLE OF CISA.CONFIG FILE #################
        # Run second step of cisa
        python cisa.py cisa.config
```
